# *SL-quant:* a fast and flexible pipeline to quantify spliced leader trans-splicing events from RNA-seq data

**DOI:** 10.1093/gigascience/giy084

**Published:** 2018-07-11

**Authors:** Carlo Yague-Sanz, Damien Hermand

**Affiliations:** URPhyM-GEMO, The University of Namur (UNamur), 61 rue de Bruxelles, 5000 Namur, Belgium

**Keywords:** NGS, RNA-seq, maturation, trans-splicing, sequence analysis

## Abstract

**Background:**

The spliceosomal transfer of a short spliced leader (SL) RNA to an independent pre-mRNA molecule is called SL trans-splicing and is widespread in the nematode *Caenorhabditis elegans*. While RNA-sequencing (RNA-seq) data contain information on such events, properly documented methods to extract them are lacking.

**Findings:**

To address this, we developed *SL-quant*, a fast and flexible pipeline that adapts to paired-end and single-end RNA-seq data and accurately quantifies SL trans-splicing events. It is designed to work downstream of read mapping and uses the reads left unmapped as primary input. Briefly, the SL sequences are identified with high specificity and are trimmed from the input reads, which are then remapped on the reference genome and quantified at the nucleotide position level (SL trans-splice sites) or at the gene level.

**Conclusions:**

*SL-quant* completes within 10 minutes on a basic desktop computer for typical *C. elegans* RNA-seq datasets and can be applied to other species as well. Validating the method, the SL trans-splice sites identified display the expected consensus sequence, and the results of the gene-level quantification are predictive of the gene position within operons. We also compared *SL-quant* to a recently published SL-containing read identification strategy that was found to be more sensitive but less specific than *SL-quant*. Both methods are implemented as a bash script available under the MIT license [1]. Full instructions for its installation, usage, and adaptation to other organisms are provided.

## Background

**Figure 1:**
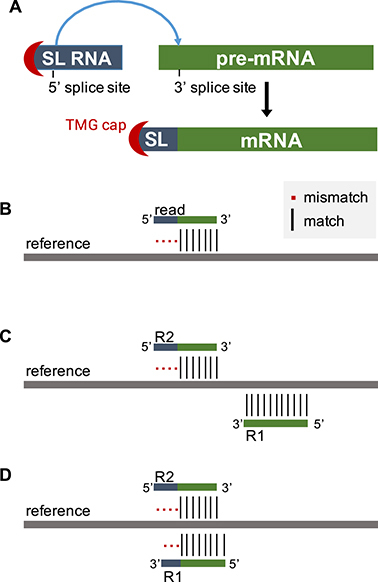
Trans-splicing and RNA-seq. (**A**) The trans-splicing process. Splice leader RNA precursors (SL RNA) are small nuclear RNAs capped with a trimethyl-guanosine (TMG). The 5’-region of the SL RNA, including the TMG cap, is spliced on the first exon of the pre-mRNAs. (**B**) Reads originating from trans-spliced RNA fragments do not map end-to-end to the reference genome. (**C**) The left-most read (R2) of a read pair does not map end-to-end to the reference. (**D**) Special case when the paired-end reads “dovetail” and both reads do not map end-to-end to the reference due to the SL sequence.

The capping, splicing, and polyadenylation of eukaryotic pre-mRNAs are well-studied maturation processes that are essential for proper gene expression in eukaryotes [2]. Much less is known about spliced leader (SL) trans-splicing, a process by which a capped small nuclear RNA called spliced leader is spliced onto the 5’ end of a pre-mRNA molecule, substituting for canonical capping [3] (Fig. 1A). SL trans-splicing has a patchy phylogenetic distribution ranging from protists [4] to bilaterian metazoans, including nematodes, rotifers [5], and even chordates [6]. It appears not conserved in mammals, although “non-SL” trans-splicing events—when exons from two different RNA transcripts are spliced together—have been detected at low frequency [7]. In contrast, SL trans-splicing is widespread in the *Caenorhabditis elegans* nematode where there are two classes of SL, SL1 and SL2, which trans-splice about 70% of the mRNA transcripts. Strikingly, the SL2 trans-splicing is highly specific for genes in position two and over within operons that range from two to eight genes expressed from a single promoter [8].

While the function of SL trans-splicing begins to be elucidated [9], its regulation remains unclear. To study this question, two main strategies have been proposed to exploit RNA-sequencing (RNA-seq) data in order to quantify SL trans-splicing. The first one involves the mapping of the reads to a complex database containing all the possible trans-spliced gene models [10, 11]. The creation of such a database requires the *in silico* trans-splicing of every SL sequence isoform (12 in *C. elegans*) to all the putative trans-splice sites predicted for a gene. In contrast, the second strategy does not rely on trans-splice site annotation or prediction. Instead, the SL sequences are directly identified in reads partially mapped to the genome or transcriptome [12-14]. However, no implementation of these methods is directly available, which prompted us to develop, test, and optimize *SL-quant*, a ready-to-use pipeline that applies the second strategy to rapidly quantify SL trans-splicing events from RNA-seq data.

### Pipeline overview

In order to search for SL sequences in a limited number of reads, only unmapped reads are used as input for *SL-quant*, assuming that reads containing the SL sequence (or the 3’ end of it) would not map on the reference genome or transcriptome (Fig. 1B). This implies that a first round of mapping must precede the use of *SL-quant*. It must be performed end-to-end in order to guarantee that reads originating from trans-spliced RNA fragments do not map. In addition to this specification, any bam file containing unmapped reads can be fed into *SL-quant*, making it particularly well suited for subsequent analyses of previously generated data.

In the case paired-end reads are available, only the unmapped reads originating from the left-most ends of the fragments are considered. In addition, we developed an optimized paired-end mode (*-p –paired* option) that further limits the search for SL-containing reads by filtering out the unmapped reads whose mates are also unmapped. This assumes that only the left-most read of a pair originating from a trans-spliced fragment would not map due to the SL sequence while the other one would map (Fig. 1C). This is generally true unless the fragment is so small that the mates significantly overlap with each other (Fig. 1D).

**Figure 2:**
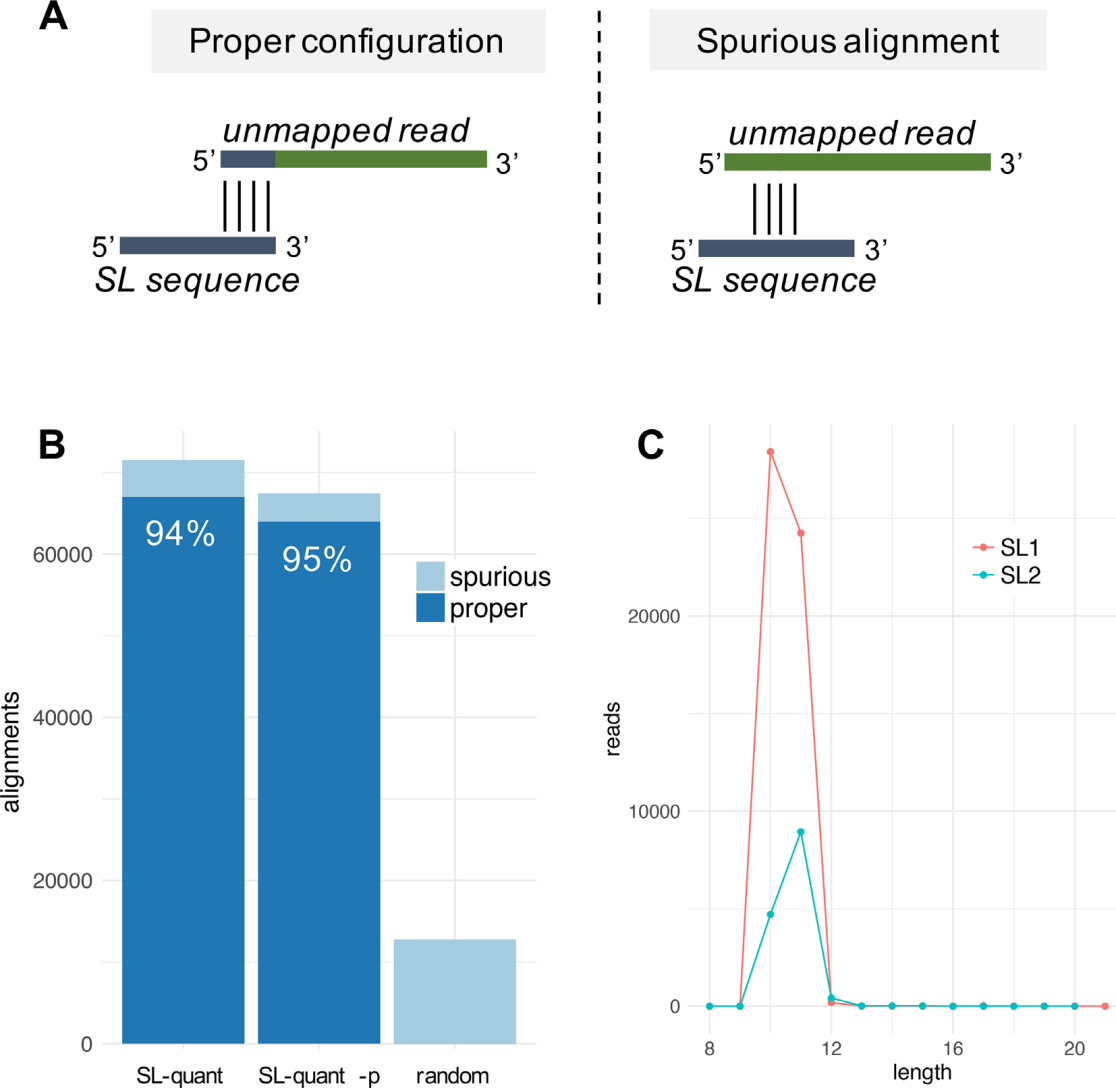
Configuration of the BLAST alignments. **(A)** In*SL-quant*, the BLAST alignments are considered as properly configured if starting from the 5’ end of the unmapped read and ending at the 3’ end of the SL sequence. **(B)** Proportion of properly configured alignments out of the significant alignment identified by *SL-quant* in single and paired-end (-p) mode on the *SRR1585277* dataset, or on 10^6^ random reads in single-end mode. **(C)** Number of properly configured significant alignments found by *SL-quant* on the *SRR1585277* dataset (single-end mode) by alignment length on the SL1 or SL2 sequences.

To identify SL trans-splicing events, the input reads are aligned locally to the SL sequences with Basic Local Alignment Search Tool (BLAST) [15]. Reads whose 5’ end belongs to a significant alignment (e-value <5%) that covers the 3΄ end of the SL sequence (Fig. 2A, left panel) are considered SL-containing reads. Then, the SL-containing reads are trimmed of the SL sequence (based on the length of the BLAST alignment) and mapped back on the *C. elegans* genome with HISAT2 [16]. Finally, the remapped reads are counted at the gene level with *featureCounts* [17] to obtain a quantification of the SL1 and SL2 trans-splicing events per genes.

### SL-containing reads identification

We tested *SL-quant* on the single-end modENCODE_4594 [18] dataset (2.5 × 10^6^ unmapped reads) and the paired-end SRR1585277 [19] dataset (1.3 × 10^6^ unmapped left reads) using a desktop computer with basic specifications. Every run was completed within 10 minutes using four threads, with a processing rate of about 10^6^ unmapped reads by 5 minutes.

In order to assess the specificity of the BLAST alignments, we reasoned that reads originating from a trans-spliced RNA would align to the 3’ end of the SL sequence from their 5’ end, while random alignment would start anywhere (Fig. 2A). The fact that 94% of significant alignments were in that specific configuration indicates good specificity (Table 1 and Fig. 2B). In contrast, we obtained less than 0.3% with randomly generated reads. In paired-end mode, fewer alignments were found, but a slightly higher proportion of them (95%) were in proper configuration and considered SL-containing reads. This was expected given the more stringent prefiltering implemented in that mode. When considering only the nonsignificant alignments, we obtained intermediate proportions of proper configuration (15%–20%), suggesting that most, but not all, of those nonsignificant alignments were spurious.

**Table 1:** Identification of SL-containing reads by *SL-quant*

				Significant alignments		Nonsignificant alignments	
Dataset	Method	Total reads	Input reads	Total	Properly configured	Total	Properly configured
SRR1585277	SL-quant	40 × 10^6^	1.3 × 10^6^	71, 512	67,021 (94%)	70 211	10,359 (15%)
	SL-quant -p	40 × 10^6^	0.9 × 10^6^	67, 463	64,010 (95%)	47 596	9,849 (21%)
modENCODE_4594	SL-quant	30 × 10^6^	2.5 × 10^6^	168, 351	158,529 (94%)	100 139	20,417 (20%)
random	SL-quant	1 × 10^6^	1.0 × 10^6^	12, 788	36 (0.3%)	43 501	83 (0.2%)

SL-containing reads are defined as reads with significant and properly configured alignment to the SL sequences (sixth column).

Despite the *C. elegans* SL sequences being 22 nucleotides (nt) long, most alignments cover them on only 10–11 nt (Fig. 2C), with a preference for 10 nt alignment for SL1-containing reads and 11 nt alignments for SL2-containing reads. This could be caused by reverse transcriptase drop-off during the library preparation due to secondary structure and the proximity of the hypermethylated cap at the 5’ end of the SL. Moreover, in classic RNA-seq library preparation protocols, the second-strand synthesis is primed by RNA oligonucleotides generated by the digestion of the RNA-DNA duplex obtained after the first strand synthesis. This results in truncated dsDNA fragments that do not preserve the 5’ end of the original RNA fragments [20].

### SL trans-splice sites identification

**Figure 3:**
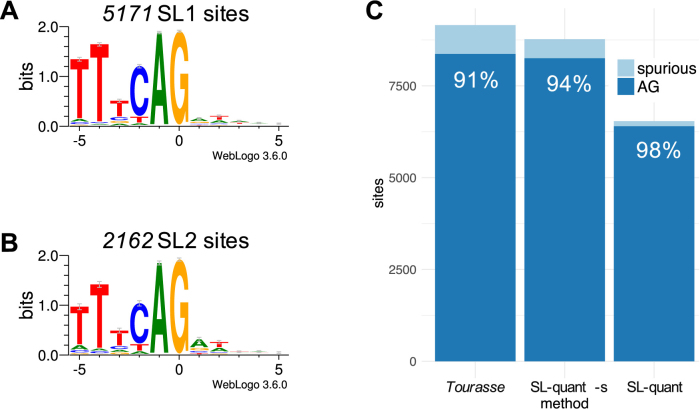
SL-sites consensus sequence. **(A)** Sequence logo of the sequence environment surrounding SL1 or **(B)** SL2 trans-splice sites determined by *SL-quant* on the *SRR1585277* dataset in single-end mode. **(C)** Proportion of AG sequences in SL trans-splice sites identified by *SL-quant* on the *SRR1585277* dataset with the method used in Tourasse et al. 2017 [14] and with *SL-quant* in single-end mode with or without the sensitive option (-s).

While we designed SL-quant with the idea of quantifying SL trans-splicing events by gene, it is also possible to use it to identify the 3’ trans-splice sites at single-nucleotide resolution. SL trans-splice sites are known to display the same UUUCAG consensus as *cis*-splice sites [21], which could be verified with our method (Fig. 3A, 3B). Previous work described a significant switch from A to G after consensus sequence (position +1) for the SL1 trans-splice sites compared to SL2 trans-splice sites [21]. At that position, we observed a decreased preference for A for the SL1 trans-splice sites, but no significant enrichment in G. This discrepancy could be due to the fact that we identified (and included in the consensus) about 20 times more SL1 trans-splice sites than previously reported.

As SL trans-splice sites (and splice sites in general) contain an almost invariant AG sequence, we reasoned that non-AG splice sites were potential “spurious” trans-splice sites. In order to assess the performances of our method, we considered identified sites bearing the “AG” consensus as true positives (TPs). Reciprocally, we considered any other sites identified as false positives (FPs), although we cannot completely exclude the existence of nonconsensus splice sites. These reasonable approximations allow us to characterize our method despite not knowing the ground truth. Indicating excellent specificity (ability to exclude FP), 98% of the sites identified by *SL-quant* display the AG consensus, regardless of the mode used (single or paired) and the dataset studied (Table 2).

**Table 2:** Performances of *SL-quant* with various parameters.

Dataset	Method	Run time	Mapped SL-containing reads	Trans-splice sites	Site is “AG” consensus (%)
SRR1585277	*SL-quant*	4 minutes 02 seconds	65,126	6,301	6,149 (98)
	*SL-quant* -p	5 minutes 14 seconds	61,451	6,539	6,402 (98)
	*SL-quant* -s	2 minutes 45 seconds	120,542	8,770	8,254 (94)
	*SL-quant* -s -p	6 minutes 58 seconds	114,948	8,436	7,957 (94)
	*Tourasse*	4 minutes 45 seconds	120,710	8,932	8,260 (92)
modENCODE_4594	*SL-quant*	9 minutes 51 seconds	146,358	8,247	8,081 (98)
	*SL-quant* -s	3 minutes 10 seconds	258,706	10,735	9,948 (93)
	*Tourasse*	5 minutes 08 seconds	259,284	11,155	9,953 (89)
random	*SL-quant*	3 minutes 20 seconds	53	52	34 (65)
	*SL-quant* -s	1m23s	5,757	5,692	5,612 (99 ^a^)
	*Tourasse*	2m24s	8,890	8,777	5,612 (64)

a The very high proportion of “AG” sites for the random dataset is an artifact caused by the fact that the reads were generated from randomly sampling the genome and that all the *C. elegans* SL sequences end by AG. -p: paired-end mode; -s: sensitive mode.

### Comparison with a previous method

We also compared our method with a re-implementation of the SL-containing read identification strategy previously reported [14]. Briefly, the unmapped reads whose 5’ end align to the SL sequences (or their reverse complement) on at least 5 nt with at most 10% mismatch are considered SL-containing reads. The alignment is realized with *cutadapt* [22] that directly trims the SL sequences from the unmapped reads so they can be remapped to the genome.

Compared to *SL-quant*, this conceptually similar method was faster and identified almost twice the number of SL-containing reads from the real datasets and 150 times the number of SL-containing reads from random reads (Table 2). More splice-sites were identified, but the proportion of spurious (nonconsensus) trans-splice sites increased almost 5-fold (Fig. 3C).

The method developed in [14] has a higher detection power but appears less specific than *SL-quant*. Nevertheless, we consider it an interesting option for applications requiring more sensitivity (ability to detect TP) than specificity. Therefore, we decided to re-implement it within *SL-quant* as an *[-s –sensitive]* option with the following enhancement:

- The input reads, if strand specific, are aligned to the SL sequences only (not their reverse complement).
- With paired-end data in single-end mode, only the left-most unmapped reads are considered as input.
- With paired-end data in paired-end mode, only the left-most unmapped reads whose mates are mapped are considered as input.

These modifications significantly improved the specificity of the method (although not to the level of *SL-quant*), with almost no compromise on sensitivity regarding SL trans-splice site detection (Fig. 3C) or SL-containing read identification (Table 2).

### Gene-level quantification

**Figure 4:**
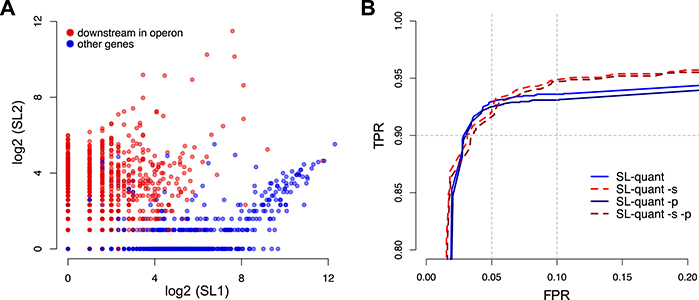
Prediction of gene position in operons. **(A)** Number of SL1 and SL2 trans-splicing events by genes as calculated using *SL-quant*. Genes annotated as downstream in the operons are represented as red dots. **(B)** Receiver operating characteristic curve analysis using the SL2/(SL1 + SL2) ratio as a predictor of downstream position in operons for the 5,521 genes with at least one trans-splicing event detected. The number of SL1 and SL2 trans-splicing events by genes was calculated using *SL-quant* in single or paired (-p) mode, with or without the sensitive (-s) option. TPR: true-positive rate, FPR: false-positive rate.

Finally, we tested *SL-quant* for its ability to predict gene position within operons as SL2-trans-splicing is the best predictor of transcription initiated upstream of another gene [11] (Fig. 4A). Using the ratio of *SL2/(SL1 + SL2)* from the *SL-quant* output as a predictor of gene positions in operons, receiver operating characteristic curve analysis reveals a high TP rate (>90%) at a 5% false discovery rate threshold, regardless of *SL-quant* options (Fig. 4B). However, when tolerating more FPs, *SL-quant* in *sensitive* mode is a superior predictor.

### Conclusion

**Figure 5:**
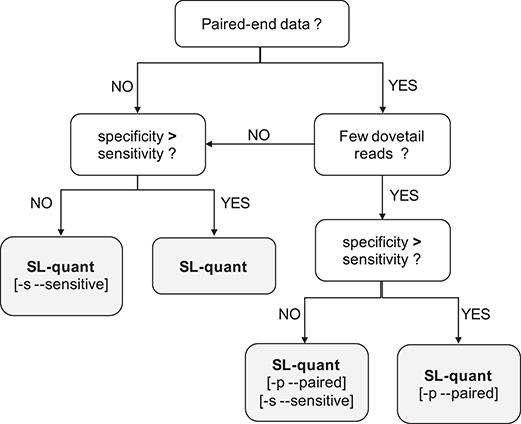
Recommendations on *SL-quant* usage. [-s –sensitive]: it provides increased detection power at the cost of some specificity and it is significantly faster. It is not recommended for applications that are very sensitive to FPs (e.g., trans-splice sites detection) but is an interesting option otherwise (e.g., gene-level quantification of SL trans-splicing events). [-p –paired]: a more stringent prefiltering reduces the number of reads aligned to the SL sequences. It can only be used with paired-end reads. It is not recommended when the average fragment size is small (many “dovetail” reads). It can be used in combination with the [-s –sensitive] option.

In summary, *SL-quant* is able to rapidly and accurately quantify trans-splicing events from RNA-seq data. It comes as a well-documented and ready-to-use pipeline in which two main options were implemented to fit the type of input data and the intended usage of the quantification (Fig. 5). Importantly, this work provides a way to test and validate SL trans-splicing quantification methods that might serve as a baseline for future development of such methods.

Recently, the hypothesis that the SL trans-splicing mechanism originates from the last eukaryotic common ancestor has been proposed to explain its broad phylogenetic distribution [23]. Given the number of applicable species, the continuously decreasing cost of RNA-seq experiments, and the thinner line between model and nonmodel organisms, it is likely that the SL trans-splicing will be studied in a growing number of species. Therefore, a procedure to adapt *SL-quant* to species other than *C. elegans*, requiring only a few steps, is detailed online. As a proof of concept, we successfully applied *SL-quant* to six additional RNA-seq libraries from five species (Table 3). In the near future, we anticipate that the application of *SL-quant* to various datasets might become instrumental in unveiling trans-splicing regulation in the model organism *C. elegans* and other organisms.

**Table 3:** *SL-quant* can be applied to a wide range of datasets from various species, with varying read length and made with various library preparation protocols.

Organism	Dataset	Read length (nt)	Total reads	Input reads	Mapped SL-containing reads	Trans-splice sites (% AG)
*Caenorhabditis elegans*	SRR1585277	76	40 × 10^6^	1.3 × 10^6^	120,542	8,770 (94)
	modENCODE_4594	76	30 × 10^6^	2.5 × 10^6^	258,706	10,735 (93)
	SRR2832497 (*)	41	4 × 10^6^	1.8 × 10^6^	16,307	4,882 (87)
*Caenorhabditis briggsae*	SRR440441	42	11 × 10^6^	5.7 × 10^6^	117,738	8,382 (93)
	SRR440557	42	12 × 10^6^	4.8 × 10^6^	176,205	11,495 (92)
*Caenorhabditis brenneri*	modENCODE_4705	76	4 × 10^6^	0.4 × 10^6^	74,689	8,891 (97)
*Caenorhabditis* *remanei*	modENCODE_4206	76	9 × 10^6^	1.8 × 10^6^	248,335	11,223 (92)
*Trypanosoma brucei*	SRR038724	35	8 × 10^6^	2.2 × 10^6^	40,320	6,703 (89)

The datasets modENCODE_4594, SRR2832497, and SRR038724 are single end, the others are paired. The asterisk (*) for the SRR2832497 denotes that the second-strand synthesis was made using a ligation-based protocol instead of the classic random priming protocol. All datasets were analyzed with the same *SL-quant* parameters: single-end mode with the -s –sensitive option

## Methods

We ran *SL-quant* with four threads (default) on the modENCODE_4594, modENCODE_4705, modENCODE_4206 [18], SRR2832497 [24], SRR440441, SRR440557 [25], SRR038724 [26], and SRR1585277 [19] poly-A + datasets using a desktop computer with a 2.8-GHz processor and 8 GB random access memory. The *C. elegans, C. briggsae, C. brenneri, and C. remanei* reference genome and annotation (WS262) were downloaded from wormbase [27]. The *T. brucei* reference genome and annotation (Apr_2005 version) were downloaded from Ensembl [28]. The read mapping steps prior to using *SL-quant* and at the end of the pipeline were performed using *HISAT2* [16] (v 2.0.5) with parameters –no-softclip –no-discordant –min-intronlen 20 –max-intronlen 5000. As we noticed adaptor contamination in the modENCODE_4594 dataset, *trimmomatic* [29] (v 0.36) was used to trim them off prior to the mapping. *Samtools* [30] (v 1.5), *picard* [31] (v 2.9), and *bedtools* [32] (v 2.26) were used to convert and/or filter the reads at various stages of the pipeline. BLAST+ (v 2.6) [15] was used to align the reads locally to the relevant SL sequences [33, 34] with parameter -task blastn -word_size 8 max_target_seqs 1. Alternatively, *cutadapt* (v 1.14) [22] was used to directly trim the SL sequences from the reads with parameters -O 5 -m 15 –discard-untrimmed. *FeatureCounts* [17] was used to summarize re-mapped SL-containing reads at the gene level. *Bedtools* [32] was used to summarize mapped SL-containing reads at the genomic position level and to generate random reads by randomly sampling the *C. elegans* genome for 50-nt segments. Sequence logo were made with *weblogo* [35]. Finally, R [36] (v 3.4) was used for analyzing and visualizing the data.

## Availability of source code and requirements

Project name: SL-quant

Project home page: https://github.com/cyaguesa/SL-quant

Operating system(s): UNIX-based systems (tested on macOS 10.12.6, macOS 10.11.6, Ubuntu 14.04)

Programming language: Shell, R

Other requirements: The BLAST+ suite (2.6.0 or higher), samtools (1.5 or higher), picard-tools (2.9.0 or higher), featureCounts from the subread package. (1.5.0 or higher), bedtools (2.26.0 or higher), cutadapt (1.14 or higher), hisat2 (2.0.5 or higher). Installation instruction for those requirements is provided online.

License: MIT

RRID:SCR_016205

## Supplementary Material

GIGA-D-18-00139_(Original_Submission).pdf

GIGA-D-18-00139_Revision_1.pdf

Response_to_Reviewer_Comments_Original_Submission.pdf

Reviewer_1_Report_(Original_Submission) -- Wenfeng Qian, PhD 5/20/2018 Reviewed

Reviewer_1_Report_(Revision_1) -- Wenfeng Qian, PhD 6/8/2018 Reviewed

Reviewer_2_Report_(Original_Submission) -- Serghei Mangul 05/22/2018 Reviewed

Reviewer_2_Report_(Revision_1) -- Serghei Mangul 6/16/2018 Reviewed

Reviewer_3_Report_(Original_Submission) -- Ben Langmead 22 May 2018 Reviewed

Reviewer_3_Report_(Revision_1) -- Ben Langmead 6/12/2018 Reviewed

Supplemental Figure

## Availability of supporting data

The datasets supporting the results presented here are available in the modMine or the European Nucleotide Archive (ebi-ENA) repositories, under the identifiers modENCODE_4594, modENCODE_4705, modENCODE_4206, SRR1585277, SRR2832497, SRR440441, SRR440557, SRR038724. Snapshots of the code and other supporting data are available in the *GigaScience* repository, GigaDB [1].

## Additional file

**Figure S1. (A)** Number of properly oriented significant alignments found by SL-quant on the *SRR2832497* dataset (single-end mode) by alignment length on the SL1 or SL2 sequences. **(B)** Number of properly oriented significant alignments found by with the method used in *Tourasse et al, 2017* on the *SRR1585277* dataset by alignment length on the SL1 or SL2 sequences.

## Abbreviations

BLAST: Basic Local Alignment Search Tool; FP: false positive; nt: nucleotide; RNA-seq: RNA sequencing; SL: spliced leader; TP: true positive.
