# Supplementary material for: SL-quant: a fast and flexible pipeline to quantify spliced leader trans-splicing events from RNA-seq data
Source: Gigascience. 2018 Jul 11;7(7):giy084. doi: 10.1093/gigascience/giy084 (PMC6055573; doi:10.1093/gigascience/giy084)

GigaScience

SL-quant: A fast and flexible pipeline to quantify spliced leader trans-splicing events from RNA-seq data.

--Manuscript Draft--

**Manuscript Number:**	GIGA-D-18-00139
**Full Title:**	SL-quant: A fast and flexible pipeline to quantify spliced leader trans-splicing events from RNA-seq data.
**Article Type:**	Technical Note
**Funding Information:**	Fonds pour la Formation à la Recherche dans l'Industrie et dans l'Agriculture — Mr Carlo Yague-Sanz
**Abstract:**	Background<br>The spliceosomal transfer of a short spliced leader (SL) RNA to an independent pre-mRNA molecule is called SL trans-splicing and is widespread in the nematode C. elegans. While RNA-seq data contain information on such events, properly documented methods to extract them are lacking.<br><br>Findings<br>To address this, we developed SL-quant, a fast and flexible pipeline that adapts to paired-end and single-end RNA-seq data and accurately quantifies SL trans-splicing events. It is designed to work downstream of read mapping and uses the reads left unmapped as primary input. Briefly, the SL-sequences are identified with high specificity and are trimmed from the input reads, which are then re-mapped on the reference genome and quantified at the nucleotide position level (SL trans-splice sites) or at the gene level.<br><br>Conclusions<br>SL-quant completes within 10 minutes on a basic desktop computer for typical RNA-seq datasets. Validating the method, the SL trans-splice sites identified display the expected consensus sequence and the results of the gene-level quantification are predictive of the gene position within operons. We also compared SL-quant to a recently published SL-containing read identification strategy which revealed being more sensitive, but less specific than SL-quant. Both methods are implemented as a bash script available under the MIT licence at https://github.com/cyaguesa/SL-quant. Full instructions for its installation, usage, and adaptation to other organisms are provided.
**Corresponding Author:**	Carlo Yague-Sanz, M.D.<br>Université de Namur<br>Namur, BELGIUM
**Corresponding Author Secondary Information:**	
**Corresponding Author's Institution:**	Université de Namur
**Corresponding Author's Secondary Institution:**	
**First Author:**	Carlo Yague-Sanz, M.D.
**First Author Secondary Information:**	
**Order of Authors:**	Carlo Yague-Sanz, M.D.<br>Damien Hermand, PhD
**Order of Authors Secondary Information:**	
**Additional Information:**	
**Question**	**Response**
Are you submitting this manuscript to a special series or article collection?	No

Powered by Editorial Manager® and ProduXion Manager® from Aries Systems Corporation

**Experimental design and statistics**<br><br>Full details of the experimental design and statistical methods used should be given in the Methods section, as detailed in our Minimum Standards Reporting Checklist. Information essential to interpreting the data presented should be made available in the figure legends.<br><br>Have you included all the information requested in your manuscript?	Yes
**Resources**<br><br>A description of all resources used, including antibodies, cell lines, animals and software tools, with enough information to allow them to be uniquely identified, should be included in the Methods section. Authors are strongly encouraged to cite Research Resource Identifiers (RRIDs) for antibodies, model organisms and tools, where possible.<br><br>Have you included the information requested as detailed in our Minimum Standards Reporting Checklist?	Yes
**Availability of data and materials**<br><br>All datasets and code on which the conclusions of the paper rely must be either included in your submission or deposited in publicly available repositories (where available and ethically appropriate), referencing such data using a unique identifier in the references and in the "Availability of Data and Materials" section of your manuscript.<br><br>Have you have met the above requirement as detailed in our Minimum Standards Reporting Checklist?	Yes

Manuscript Click here to download Manuscript 20180417_SL-quant.pdf

SL-quant: A fast and flexible pipeline to quantify spliced leader trans-splicing events from RNA-seq data.

Yague-Sanz Carlo (carlo.yaguesanz@unamur.be)[1*] and Hermand Damien (damien.hermand@unamur.be)[1]

[1] URPhyM-GEMO, University of Namur, 5000 Namur, Belgium.

[*] To whom correspondence should be addressed

Running head: Quantification of trans-splicing events after RNA-seq

ABSTRACT

Background

The spliceosomal transfer of a short spliced leader (SL) RNA to an independent pre-mRNA molecule is called SL trans-splicing and is widespread in the nematode *C. elegans*. While RNA-seq data contain information on such events, properly documented methods to extract them are lacking.

Findings

To address this, we developed *SL-quant,* a fast and flexible pipeline that adapts to paired-end and single-end RNA-seq data and accurately quantifies SL trans-splicing events. It is designed to work downstream of read mapping and uses the reads left unmapped as primary input. Briefly, the SL-sequences are identified with high specificity and are trimmed from the input reads, which are then re-mapped on the reference genome and quantified at the nucleotide position level (SL trans-splice sites) or at the gene level.

Conclusions

SL-quant completes within 10 minutes on a basic desktop computer for typical RNA-seq datasets. Validating the method, the SL trans-splice sites identified display the expected consensus sequence and the results of the gene-level quantification are predictive of the gene position within operons. We also compared *SL-quant* to a recently published SL-containing read identification strategy which revealed being more sensitive, but less specific than *SL-quant*. Both methods are implemented as a bash script available under the MIT licence at https://github.com/cyaguesa/SL-quant. Full instructions for its installation, usage, and adaptation to other organisms are provided.

KEYWORDS: NGS, RNA-seq, maturation, trans-splicing, sequence analysis.

FINDINGS

Background

The capping, splicing and polyadenylation of eukaryotic pre-mRNAs are well-studied maturation processes that are essential for proper gene expression in eukaryotes [1]. Much less is known about spliced leader trans-splicing, a process by which a capped small nuclear RNAs called spliced leader (SL) is spliced onto the 5'-end of a pre-mRNA molecule, substituting for canonical capping [2] **(fig 1.A)**. SL trans-splicing has a patchy phylogenetic distribution ranging from protists [3] to bilaterian metazoans, including nematodes, rotifers [4] and even chordates [5]. In the *C. elegans* nematode, there are two classes of SL, called SL1 and SL2, which trans-splice about 70% of the mRNA transcripts. Strikingly, the SL2 trans-splicing is highly specific for genes in position two and over within operons that range from two to eight genes expressed from a single promoter [6].

While the function of SL trans-splicing begins to be elucidated [7], its regulation remains unclear. To study this question, two main strategies have been proposed to exploit RNA-seq data in order to quantify SL trans-splicing. The first one involves the mapping of the reads to a complex database containing all the possible trans-spliced gene models [8, 9]. The creation of such a database requires the *in silico* trans-splicing of every SL sequence isoform (12 in *C. elegans*) to all the putative trans-splice sites predicted for a gene. In contrast, the second strategy does not rely on trans-splice site annotation or prediction. Instead, the SL sequences are directly identified in reads partially mapped to the genome or transcriptome [10-12]. However, no implementation of these methods is directly available, which prompted us to develop, test and optimize *SL-quant*, a ready-to-use pipeline that applies the second strategy to rapidly quantify SL trans-splicing events from RNA-seq data.

Pipeline overview

In order to search for SL sequences in a limited number of reads, only unmapped reads are used as input for *SL-quant*, assuming that reads containing the SL-sequence (or the 3' end of it) would not map on the reference genome or transcriptome (**figure 1.B**). This implies that a first round of mapping must precede the use of *SL-quant*. It must be performed end-to-end in order to guarantee that reads originating from trans-spliced RNA fragments do not map. Beside this specification, any bam file containing unmapped reads can be fed into *SL-quant*, making it particularly well suited for subsequent analyses of previously generated data.

In the case paired-end reads are available, only the unmapped reads originating from the left-most ends of the fragments are considered. In addition, we developed an optimized paired-end mode (*-p --paired* option) that further limits the search for SL-containing reads by filtering out the unmapped reads whose mates are also unmapped. This assumes that only the left-most read of a pair originating from a trans-spliced fragment would not map due to the SL-sequence while the other one would map (**figure 1.C**). It is generally true unless the fragment is so small that the mates significantly overlap with each other (**figure 1.D**).

To identify SL trans-splicing events, the input reads are aligned locally to the SL sequences with blast [13]. Reads whose 5' end belongs to a significant alignment (e-value < 5%) that covers the 3' end of the SL-sequence (**figure 2.A**, left panel) are considered *SL-containing reads*. Then, the SL-containing reads are trimmed of the SL-sequence (based on the length of the blast alignment) and mapped back on *C. elegans* genome with HISAT2 [14]. Finally, the re-mapped reads are counted at the gene level with *featureCounts* [15] to obtain a quantification of the SL1 and SL2 trans-splicing events per genes.

SL-containing reads identification

We tested *SL-quant* on the single-end modENCODE_4594 [16] and the paired-end SRR1585277 [17] datasets using a desktop computer with basic specifications. Every run completed within 10 min.

In order to assess the specificity of the blast alignments, we reasoned that reads originating from a trans-spliced RNA would align to the 3' end of the SL sequence from their 5' end, while random alignment would start anywhere (**figure 2.A**). The fact that 94% of significant alignments were in that specific configuration indicates good specificity (**table 1** and **figure 2.B**). In contrast, we obtained less than 0.3% with randomly generated reads. In paired-end mode, less alignments were found but a slightly higher proportion of them (95%) were in proper configuration and considered SL-containing reads. This was expected given the more stringent pre-filtering implemented in that mode. When considering only the non-significant alignments, we obtained intermediate proportions of proper configuration (15-20%), suggesting that most, but not all, of those non-significant alignments were spurious.

Despite the *C. elegans* SL sequences being 22 nucleotides (nt) long, most alignments cover them on only 10-11 nt **(figure 2.C)**, with a preference for 10 nt alignment for SL1-containg reads and 11 nt alignments for SL2-containing reads. This could be caused by reverse transcriptase drop-off during the library preparation due to secondary structure and the proximity of the hyper-methylated cap at the 5' end of the SL. Moreover, the 5' end of the RNA fragments is not preserved during the second-strand synthesis in classical RNA-seq library preparation protocols [18], resulting in truncated fragments.

SL trans-splice sites identification.

While we designed SL-quant with the idea of quantifying SL trans-splicing events by gene, it is also possible to use it to identify the 3' trans-splice sites at single nucleotide resolution. SL trans-splice sites are known to display the same UUUCAG consensus as *cis*-splice sites [19], which could be verified with our method (**figure 3.A,B**). Previous work described a significant switch from A to G after to consensus sequence (position +1) for the SL1 trans-splice sites compared to SL2 trans-splice site [19]. At that position, we observed a decreased preference for A for the SL1 trans-splice sites, but no significant enrichment in G. This discrepancy could be due to the fact that we identified (and included in the consensus) about 20 times more SL1 trans-splice sites than previously reported.

As SL trans-splice sites (and splice sites in general) contain an almost invariant AG sequence, we reasoned that non-AG splice sites were potential *'spurious'* trans-splice sites. Indicating excellent specificity, 98% of the sites identified by *SL-quant* display the AG consensus, regardless of the mode used (single or paired) and the dataset studied (**table 2**).

Comparison with a previous method

We also compared our method with a re-implementation of the SL-containing read identification strategy previously reported [12]. Briefly, the unmapped reads whose 5' end align to the SL sequences (or their reverse complement) on at least 5 nt with at most 10% mismatch are considered SL-containing reads. The alignment is realized with *cutadapt* [20] that directly trim the SL-sequences from the unmapped reads so they can be re-mapped to the genome.

Compared to *SL-quant*, this conceptually similar method was faster and identified almost twice the number of SL-containing reads from the real datasets, and 150 times the number of SL-containing reads from random reads (**table 2**). More splice-sites were identified, but the proportion of spurious (non-consensus) trans-splice sites increased almost 5-fold (**figure 3.C**).

All in all, the method developed in [12] has a higher detection power but appears less specific than *SL-quant*. Nevertheless, we consider it an interesting option for applications requiring more sensitivity than specificity. Therefore, we decided to re-implement it within *SL-quant* as an *[-s --sensitive]* option with the following enhancement:

- The input reads, if strand-specific, are aligned to the SL sequences only (not their reverse complement).
- With paired-end data in single-end mode, only the left-most unmapped reads are considered as input.
- With paired-end data in paired-end mode, only the left-most unmapped reads whose mates are mapped are considered as input.

These modifications significantly improved the specificity of the method (although not to the level of *SL-quant*) with almost no compromise on sensitivity regarding SL trans-splice sites detection (**figure 3.C**) or SL-containing reads identification (**table 2**).

Gene level quantification

Finally, we tested *SL-quant* for its ability to predict gene position within operons as SL2-trans-splicing is the best predictor of transcription initiated upstream of another gene [9] **(figure 4.A).** Using the ratio of *SL2/(SL1+SL2)* from the *SL-quant* output as a predictor of

gene positions in operons, ROC curve analysis reveals high true positive rate (>90%) at a 5% false discovery rate threshold, regardless of *SL-quant* options **(figure 4.B)**. However, when tolerating more false positives, *SL-quant* in *sensitive* mode is a superior predictor.

Conclusion

To sum up, *SL-quant* is able to rapidly and accurately quantify trans-splicing events from RNA-seq data. It comes as a well-documented and ready-to-use pipeline in which two main options were implemented to fit the type of input data and the intended usage of the quantification (**figure 5**). Importantly, this work provides means to test and validates SL trans-splicing quantification methods that might serve as a baseline for future development of such methods.

Recently, the hypothesis that the SL trans-splicing mechanism originates from the last eukaryotic common ancestor has been proposed to explain its broad phylogenetic distribution [21]. Given the number of concerned species, the continuously decreasing cost of RNA-seq experiments and the thinner line between model and non-model organisms, it is likely that the SL trans-splicing will be studied in a growing number of species. Therefore, a procedure to adapt *SL-quant* to species beyond *C. elegans*, requiring only a few steps, is detailed online. In the near future, we anticipate that the application of *SL-quant* to various datasets might become instrumental in unveiling trans-splicing regulation in the model organism *C. elegans* and beyond.

METHODS

We ran *SL-quant* on the modENCODE_4594 [16] and the SRR1585277 [17] datasets using a desktop computer with a 2.8 GHz processor and 8 GO RAM. The *C. elegans*

reference genome and annotation (WS262) were downloaded from wormbase [22]. The read mapping steps prior to using *SL-quant* and at the end of the pipeline were performed using *HISAT2* [14] (v 2.0.5) with parameters --no-softclip --no-discordant --min-intronlen 20 --max-intronlen 5000. As we noticed adaptor contamination in the modENCODE_4594 dataset, *trimmomatic* [23] (v 0.36) was used to trim them off prior to the mapping. *Samtools* [24] (v 1.5), *picard* [25] (v 2.9) and *bedtools* [26] (v 2.26) were used to convert and/or filter the reads at various stages of the pipeline. *Blastn* (v 2.6), from the blast+ suite [13] was used to align the reads locally to the SL sequences with parameter -word_size 8 max_target_seqs 1. Alternatively, *cutadapt* (v 1.14) [20] was used to directly trim the SL sequences from the reads with parameters -O 5 -m 15 --discard-untrimmed. *FeatureCounts* [15] was used to summarize re-mapped SL-containing reads at the gene level. *Bedtools* [26] was used to summarize mapped SL-containing reads at the genomic position level and to generate random reads by randomly sampling the *C. elegans* genome for 50 nt segments. Sequence logo were made with *weblogo* [27]. Finally, R [28] (v 3.4) was used for analysing and visualizing the data.

Availability of supporting source code and requirements

Project name: SL-quant

Project home page: https://github.com/cyaguesa/SL-quant

Operating system(s): UNIX-based systems (tested on macOS 10.12.6, macOS 10.11.6, Ubuntu 14.04)

Programming language: Shell, R.

Other requirements: blastn from the blast+ suite (2.6.0 or higher), samtools (1.5 or higher), picard-tools (2.9.0 or higher), featureCounts from the subread package. (1.5.0 or higher), bedtools (2.26.0 or higher), cutadapt (1.14 or higher), hisat2 (2.0.5 or higher). Installation instruction for those requirements is provided online.

License: MIT

RRID: SCR_016205

Availability of supporting data

The data sets supporting the results of this article are available in the modMine and the European Nucleotide Archive (ebi-ENA) repositories, under their respective identifiers of modENCODE_4594 and the SRR1585277.

DECLARATIONS

List of abbreviations:

NS: non-significant

nt: nucleotide

RNA: ribonucleic acid

ROC: receiver operatic characteristic

SL: spliced leader

Ethics approval and consent to participate: **NA**

Competing interests: The authors declare that they have no competing interests.

Consent for publication: **NA**

Funding*:* This work was supported by the FNRS-FRIA.

Authors' contributions: C.Y. designed, implemented and tested the pipeline. D.H. and C.Y. wrote the manuscript. D.H. supervised the project.

Acknowledgements: We thank Olivier Finet and Fanélie Bauer for critical reading of the manuscript.

TABLES

dataset	method	run time	input reads	significant alignments		NS alignments	
				**total**	**properly configured**	**total**	**properly configured**
SRR1585277	SL-quant	4m2s	1.3x 10^6	71512	67021 (94%)	70211	10359 (15%)
	SL-quant -p	5m14s	0.9x 10^6	67463	64010 (95%)	47596	9849 (21%)
modENCODE_4594	SL-quant	9m51	2.5x 10^6	168351	158529 (94%)	100139	20417 (20%)
random	SL-quant	3m20	1.0x 10^6	12788	36 (0.3%)	43501	83 (0.2%)

Table 1. Identification of SL-containing reads by *SL-quant*. SL-containing reads are defined as reads with significant and properly configured alignment to the SL sequences (6th column). NS: non-significant.

**dataset**	**method**	**run time**	**mapped SL-containing reads**	**trans-splice sites**	**site is "AG" consensus (%)**
SRR1585277	SL-quant	4m02s	65126	6301	6402 (98%)
	SL-quant -p	5m14s	61451	6539	6149 (98%)
	SL-quant -s	2m45s	120542	8770	8254 (94%)
	SL-quant -s -p	6m58s	114948	8436	7957 (94%)
	Tourasse	4m45s	120710	8932	8260 (92%)
modENCODE_4594	SL-quant	9m51s	146358	8247	8081 (98%)
	SL-quant -s	3m10s	258706	10735	9948 (93%)
	Tourasse	5m08s	259284	11155	9953 (89%)
random	SL-quant	3m20s	53	52	34 (65%)
	SL-quant -s	1m23s	5757	5692	5612 (99% [a])
	Tourasse	2m24s	8890	8777	5612 (64%)

Table 2. Performances of *SL-quant* with various parameters. -p: paired-end mode. -s: sensitive mode.

ENDNOTES

[a] The very high proportion of “AG” site for the random dataset is an artefact caused by the fact that the reads were generated from randomly sampling the genome and that all the *C. elegans* SL sequences end by AG.

FIGURE LEGENDS

Fig.1. Trans-splicing and RNA-seq. A) The trans-splicing process. Splice leader RNA precursors (SL RNA) are small nuclear RNAs capped with a trimethyl-guanosine (TMG). The 5’-region of the SL RNA including the TMG cap, is spliced on the first exon of the pre-mRNAs. **B)** Reads originating from trans-spliced RNA fragments do not map end-to-end to the reference genome. **C)** The left-most reads (R2) of a read pair does not map end-to-end to the reference. **D)** Special case when the paired-end reads “dovetail” and both reads do not map end-to-end to the reference due to the SL sequence.

Fig.2. Configuration of the blast alignments. A) *In SL-quant*, the blast alignments are considered as properly configured if starting from the 5’end of the unmapped read and ending at the 3’ end of the SL sequence. **B)** Proportion of properly configured alignments out of the significant alignment identified by *SL-quant* in single and paired-end (-p) mode on the *SRR1585277* dataset, or on 10^6 random reads in single-end mode. **C)** Number of properly configured significant alignments found by *SL-quant* on the *SRR1585277* dataset (single-end mode) by alignment length on the SL1 or SL2 sequences.

Fig.3. SL-sites consensus sequence. A) Sequence logo of the sequence environment surrounding SL1 or **B)** SL2 trans-splice sites determined by *SL-quant* on the *SRR1585277* dataset in single-end mode. **C)** Proportion of AG sequences in SL trans-splice sites identified

by SL-quant on the *SRR1585277* dataset with the method used in *Tourasse et al, 2017* and with *SL-quant* in single-end mode with or without the sensitive option (-s).

Fig.4. Prediction of genes position in operons. A) Number of SL1 and SL2 trans-splicing events by genes as calculated by *SL-quant*. Genes annotated as downstream in the operons are represented as red dots. **B)** Receiver operating characteristic (ROC) curve analysis using the SL2/(SL1+SL2) ratio as a predictor of downstream position in operons for the 5521 genes with at least one trans-splicing event detected. The number of SL1 and SL2 trans-splicing events by genes was calculated by *SL-quant* in single or paired (-p) mode, with or without the sensitive (-s) option. TPR: true positive rate, FPR: false positive rate.

Fig.5. **Recommendations on *SL-quant* usage.**

[-s --sensitive]: it provides increased detection power at the cost of some specificity and it is significantly faster. It is not recommended for applications that are very sensitive to false positives (e.g. trans-splice sites detection) but is an interesting option otherwise (e.g. gene level quantification of SL trans-splicing events).

[-p --paired]: a more stringent pre-filtering reduces the number of reads aligned to the SL-sequences. It can only be used with paired-end reads. It is not recommended when the average fragment size is small (many "dovetail" reads). It can be used in combination with the [-s –sensitive] option.

REFERENCES

1. Bentley DL. Coupling mRNA processing with transcription in time and space. Nature reviews Genetics. 2014;15 3:163-75. doi:10.1038/nrg3662.

2. Blumenthal T. Trans-splicing and operons in C. elegans. WormBook. 2012:1-11. doi:10.1895/wormbook.1.5.2.

3. Michaeli S. Trans-splicing in trypanosomes: machinery and its impact on the parasite transcriptome. Future Microbiol. 2011;6 4:459-74. doi:10.2217/fmb.11.20.

4. Pouchkina-Stantcheva NN and Tunnacliffe A. Spliced leader RNA-mediated trans-splicing in phylum Rotifera. Mol Biol Evol. 2005;22 6:1482-9. doi:10.1093/molbev/msi139.

5. Vandenberghe AE, Meedel TH and Hastings KE. mRNA 5'-leader trans-splicing in the chordates. Genes & development. 2001;15 3:294-303. doi:10.1101/gad.865401.

6. Blumenthal T, Evans D, Link CD, Guffanti A, Lawson D, Thierry-Mieg J, et al. A global analysis of Caenorhabditis elegans operons. Nature. 2002;417 6891:851-4. doi:10.1038/nature00831.

7. Yang YF, Zhang X, Ma X, Zhao T, Sun Q, Huan Q, et al. Trans-splicing enhances translational efficiency in C. elegans. Genome research. 2017;27 9:1525-35. doi:10.1101/gr.202150.115.

8. Hillier LW, Reinke V, Green P, Hirst M, Marra MA and Waterston RH. Massively parallel sequencing of the polyadenylated transcriptome of C. elegans. Genome research. 2009;19 4:657-66. doi:10.1101/gr.088112.108.

9. Allen MA, Hillier LW, Waterston RH and Blumenthal T. A global analysis of C. elegans trans-splicing. Genome research. 2011;21 2:255-64. doi:10.1101/gr.113811.110.

10. Maxwell CS, Antoshechkin I, Kurhanewicz N, Belsky JA and Baugh LR. Nutritional control of mRNA isoform expression during developmental arrest and recovery in C. elegans. Genome research. 2012;22 10:1920-9. doi:10.1101/gr.133587.111.

11. Boeck ME, Huynh C, Gevirtzman L, Thompson OA, Wang G, Kasper DM, et al. The time-resolved transcriptome of C. elegans. Genome research. 2016;26 10:1441-50. doi:10.1101/gr.202663.115.

12. Tourasse NJ, Millet JRM and Dupuy D. Quantitative RNA-seq meta-analysis of alternative exon usage in C. elegans. Genome research. 2017;27 12:2120-8. doi:10.1101/gr.224626.117.

13. Camacho C, Coulouris G, Avagyan V, Ma N, Papadopoulos J, Bealer K, et al. BLAST+: architecture and applications. BMC Bioinformatics. 2009;10:421. doi:10.1186/1471-2105-10-421.

14. Kim D, Langmead B and Salzberg SL. HISAT: a fast spliced aligner with low memory requirements. Nat Methods. 2015;12 4:357-60. doi:10.1038/nmeth.3317.

15. Liao Y, Smyth GK and Shi W. featureCounts: an efficient general purpose program for assigning sequence reads to genomic features. Bioinformatics. 2014;30 7:923-30. doi:10.1093/bioinformatics/btt656.

16. Gerstein MB, Lu ZJ, Van Nostrand EL, Cheng C, Arshinoff BI, Liu T, et al. Integrative analysis of the Caenorhabditis elegans genome by the modENCODE project. Science. 2010;330 6012:1775-87. doi:10.1126/science.1196914.

17. Kosmaczewski SG, Edwards TJ, Han SM, Eckwahl MJ, Meyer BI, Peach S, et al. The RtcB RNA ligase is an essential component of the metazoan unfolded protein response. EMBO Rep. 2014;15 12:1278-85. doi:10.15252/embr.201439531.

18. Agarwal S, Macfarlan TS, Sartor MA and Iwase S. Sequencing of first-strand cDNA library reveals full-length transcriptomes. Nat Commun. 2015;6:6002. doi:10.1038/ncomms7002.

19. Graber JH, Salisbury J, Hutchins LN and Blumenthal T. C. elegans sequences that control trans-splicing and operon pre-mRNA processing. RNA. 2007;13 9:1409-26. doi:10.1261/rna.596707.

20. Martin M. Cutadapt removes adapter sequences from high-throughput sequencing reads. EMBnet journal. 2011;17 1:pp. 10-2.

21. Krchnakova Z, Krajcovic J and Vesteg M. On the Possibility of an Early Evolutionary Origin for the Spliced Leader Trans-Splicing. J Mol Evol. 2017;85 1-2:37-45. doi:10.1007/s00239-017-9803-y.

22. Stein L, Sternberg P, Durbin R, Thierry-Mieg J and Spieth J. WormBase: network access to the genome and biology of Caenorhabditis elegans. Nucleic acids research. 2001;29 1:82-6.

23. Bolger AM, Lohse M and Usadel B. Trimmomatic: a flexible trimmer for Illumina sequence data. Bioinformatics. 2014;30 15:2114-20. doi:10.1093/bioinformatics/btu170.

24. Li H, Handsaker B, Wysoker A, Fennell T, Ruan J, Homer N, et al. The Sequence Alignment/Map format and SAMtools. Bioinformatics. 2009;25 16:2078-9. doi:10.1093/bioinformatics/btp352.

25. Picard tools. http://broadinstitute.github.io/picard.

26. Quinlan AR. BEDTools: The Swiss-Army Tool for Genome Feature Analysis. Curr Protoc Bioinformatics. 2014;47:11 2 1-34. doi:10.1002/0471250953.bi1112s47.

27. Crooks GE, Hon G, Chandonia JM and Brenner SE. WebLogo: a sequence logo generator. Genome research. 2004;14 6:1188-90. doi:10.1101/gr.849004.

28. R Core Team. R: A language and environment for statistical computing. Vienna, Austria: R Foundation for Statistical Computing, 2017.

figure 1

Click here to download Figure fig1_SL-quant.pdf

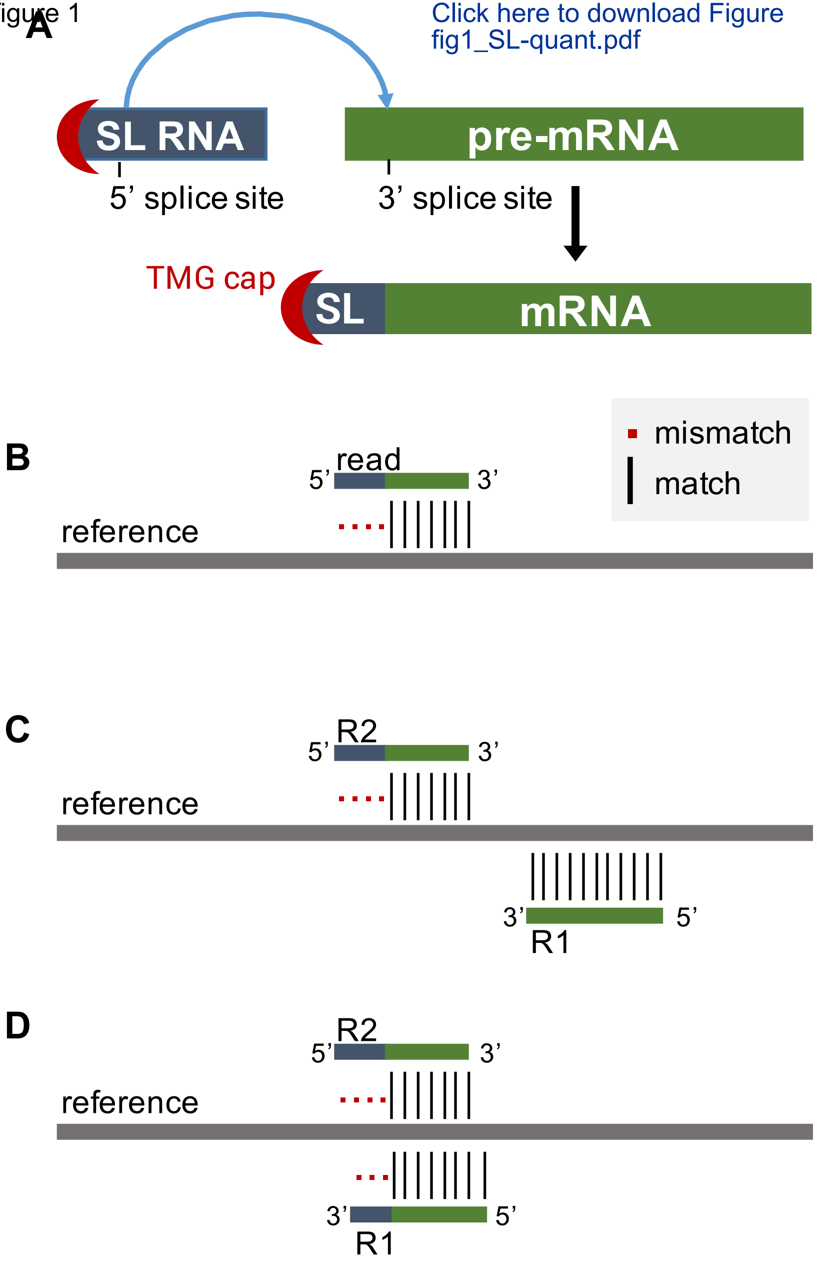

figure 2

Click here to download Figure fig2_SL-quant.pdf

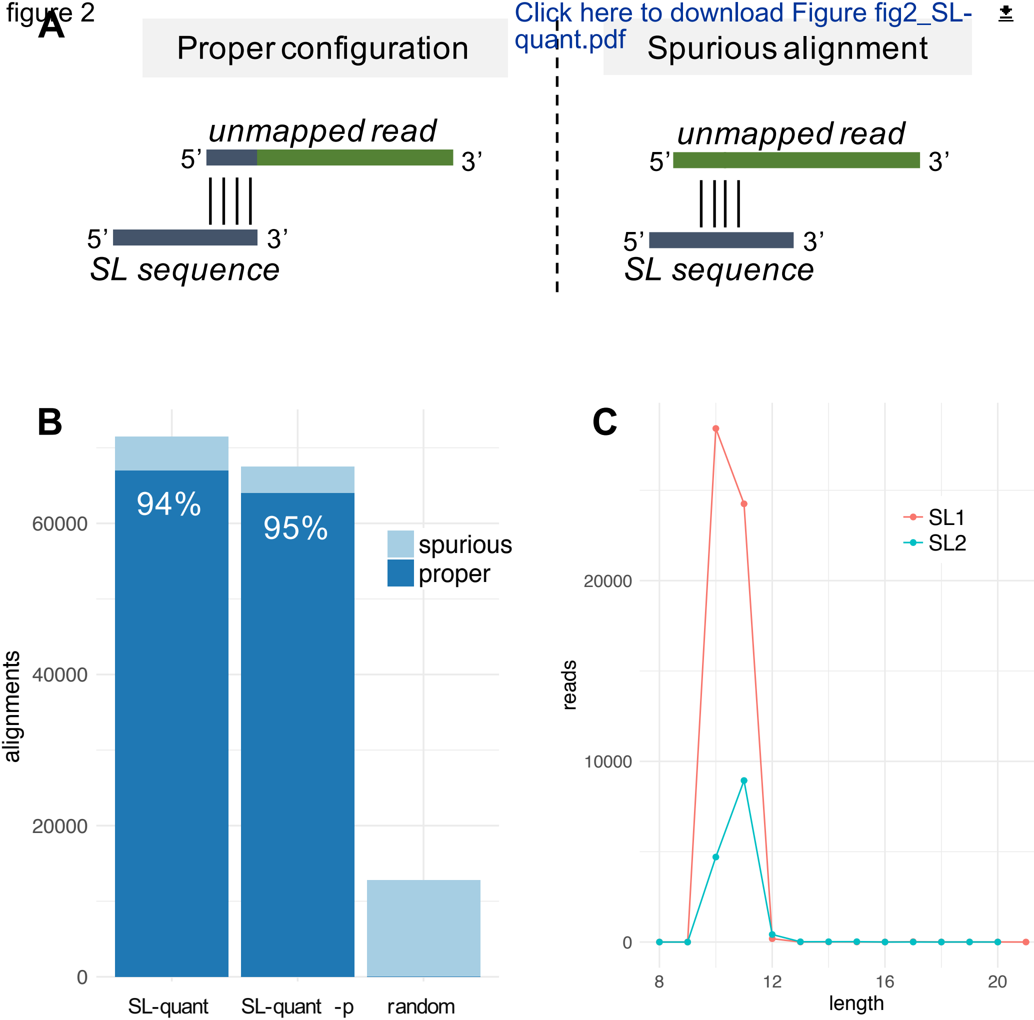

figure 3

Click here to download Figure fig3_SL-quant.pdf

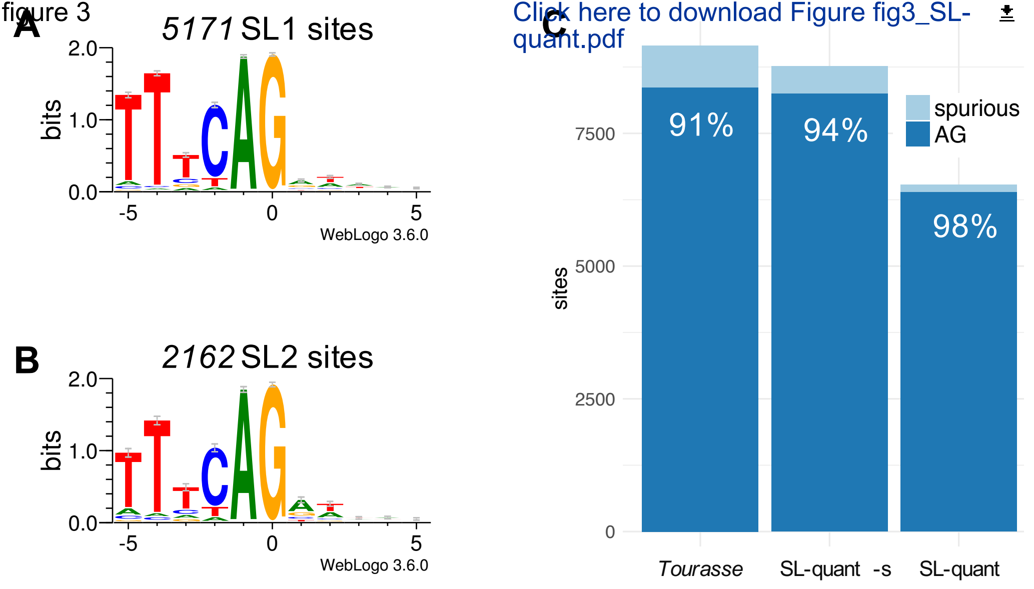

figure 4

Click here to download Figure fig4_SL-quant.pdf

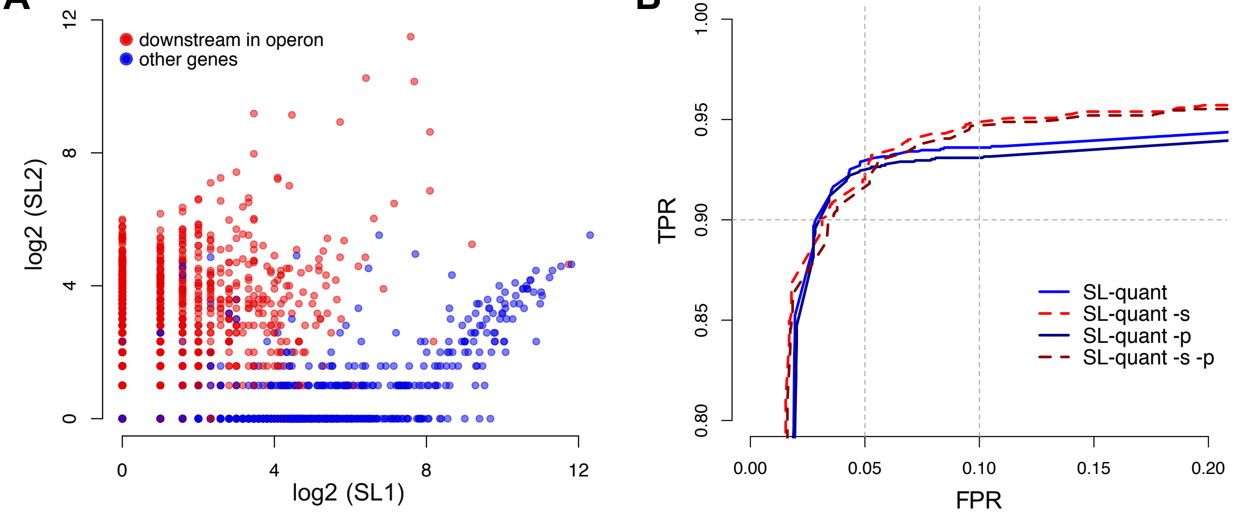

figure 5

Click here to download Figure fig5_SL-quant.pdf

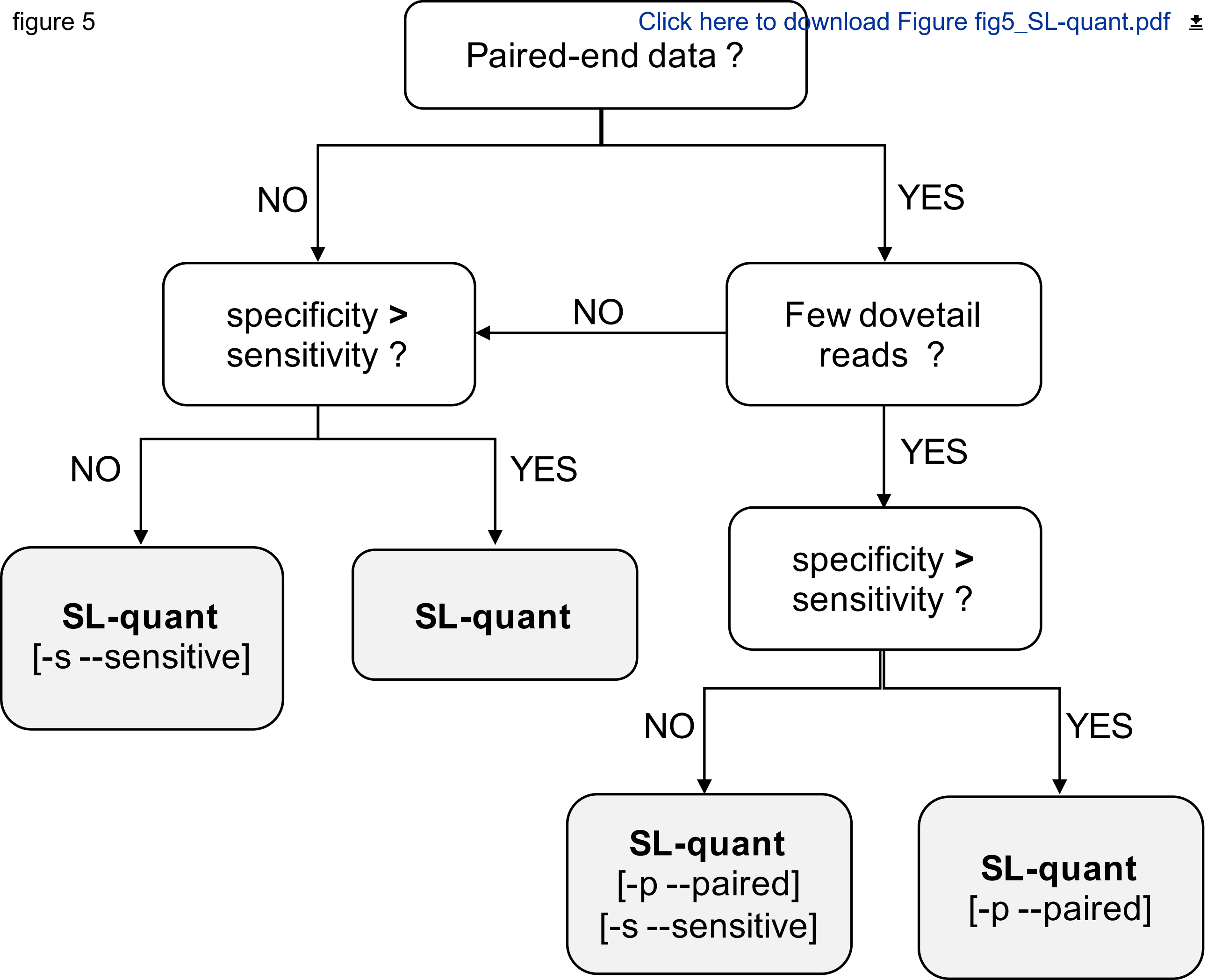

Supplement: GIGA-D-18-00139_(Original_Submission).pdf [file giy084_giga-d-18-00139_(original_submission).pdf]
