# Supplementary material for: SL-quant: a fast and flexible pipeline to quantify spliced leader trans-splicing events from RNA-seq data
Source: Gigascience. 2018 Jul 11;7(7):giy084. doi: 10.1093/gigascience/giy084 (PMC6055573; doi:10.1093/gigascience/giy084)
Supplement: GIGA-D-18-00139_Revision_1.pdf [file giy084_giga-d-18-00139_revision_1.pdf]

GigaScience

SL-quant: A fast and flexible pipeline to quantify spliced leader trans-splicing events from RNA-seq data.

--Manuscript Draft--

**Manuscript Number:**	GIGA-D-18-00139R1
**Full Title:**	SL-quant: A fast and flexible pipeline to quantify spliced leader trans-splicing events from RNA-seq data.
**Article Type:**	Technical Note
**Funding Information:**	Fonds pour la Formation à la Recherche dans l'Industrie et dans l'Agriculture — Mr Carlo Yague-Sanz
**Abstract:**	Background<br><br>The spliceosomal transfer of a short spliced leader (SL) RNA to an independent pre-mRNA molecule is called SL trans-splicing and is widespread in the nematode C. elegans. While RNA-seq data contain information on such events, properly documented methods to extract them are lacking.<br><br>Findings<br><br>To address this, we developed SL-quant, a fast and flexible pipeline that adapts to paired-end and single-end RNA-seq data and accurately quantifies SL trans-splicing events. It is designed to work downstream of read mapping and uses the reads left unmapped as primary input. Briefly, the SL-sequences are identified with high specificity and are trimmed from the input reads, which are then re-mapped on the reference genome and quantified at the nucleotide position level (SL trans-splice sites) or at the gene level.<br><br>Conclusions<br><br>SL-quant completes within 10 minutes on a basic desktop computer for typical C.elegans RNA-seq datasets, and can be applied to other species as well. Validating the method, the SL trans-splice sites identified display the expected consensus sequence and the results of the gene-level quantification are predictive of the gene position within operons. We also compared SL-quant to a recently published SL-containing read identification strategy which revealed being more sensitive, but less specific than SL-quant. Both methods are implemented as a bash script available under the MIT licence at https://github.com/cyaguesa/SL-quant. Full instructions for its installation, usage, and adaptation to other organisms are provided.
**Corresponding Author:**	Carlo Yague-Sanz, M.D.<br>Université de Namur<br>Namur, BELGIUM
**Corresponding Author Secondary Information:**	
**Corresponding Author's Institution:**	Université de Namur
**Corresponding Author's Secondary Institution:**	
**First Author:**	Carlo Yague-Sanz, M.D.
**First Author Secondary Information:**	
**Order of Authors:**	Carlo Yague-Sanz, M.D.<br>Damien Hermand, PhD
**Order of Authors Secondary Information:**	
**Response to Reviewers:**	GIGA-D-18-00139<br>SL-quant: A fast and flexible pipeline to quantify spliced leader trans-splicing events

Powered by Editorial Manager® and ProduXion Manager® from Aries Systems Corporation

from RNA-seq data.
Carlo Yague-Sanz, M.D.; Damien Hermand, PhD
GigaScience

Dear Mr Yague-Sanz,

Your manuscript "SL-quant: A fast and flexible pipeline to quantify spliced leader trans-splicing events from RNA-seq data." (GIGA-D-18-00139) has been assessed by our reviewers. Based on these reports, and my own assessment as Editor, I am pleased to inform you that it is potentially acceptable for publication in GigaScience, once you have carried out some essential revisions suggested by our reviewers.

Their reports, together with any other comments, are below. Please also take a moment to check our website at https://giga.editorialmanager.com/ for any additional comments that were saved as attachments.

In addition, please register any new software application in the SciCrunch.org database to receive a RRID (Research Resource Identification Initiative ID) number, and include this in your manuscript. This will facilitate tracking, reproducibility and re-use of your tool.

Once you have made the necessary corrections, please submit a revised manuscript online at:

https://giga.editorialmanager.com/

If you have forgotten your username or password please use the "Send Login Details" link to get your login information. For security reasons, your password will be reset.

Please include a point-by-point within the 'Response to Reviewers' box in the submission system. Please ensure you describe additional experiments that were carried out and include a detailed rebuttal of any criticisms or requested revisions that you disagreed with. Please also ensure that your revised manuscript conforms to the journal style, which can be found in the Instructions for Authors on the journal homepage.

The due date for submitting the revised version of your article is 20 Aug 2018.

We look forward to receiving your revised manuscript soon.

Best wishes,

Nicole Nogoy, Ph.D
GigaScience
www.gigasciencejournal.com

>>> We thank the editor and reviewers for their insightful and constructive comments. We agree with reviewers 1 and 2 that the method should be validated on additional datasets. We therefore applied SL-quant on six unrelated RNA-seq libraries from a total of five different organisms. The results of this new analysis are shown in a new table 3. Beside this major change, we have responded to all comments raised by the referees as detailed in the point-by-point response below. The RRID SCR_016205 has been attributed to this work and is indicated in the ‘Availability of supporting source code and requirements’ section. The revised version of this manuscript is attached with the changes to the original submission marked in red for clarity.

Reviewer reports:
-Reviewer #1: In this study, Carlo and Damien developed a fast and flexible pipeline, SL-quant, to quantify spliced leader trans-splicing events from RNA-seq data. SL trans-splicing is prevalent in nematodes. Accurate and fast identification of SL trans-splicing is of importance to understand the molecular mechanism and biological significance of SL trans-splicing. The algorithm the authors proposed in this study can be applied to

both paired-end and single-end RNA-seq data. And the pipeline accurately quantifies SL trans-splicing events.

It is overall a nice piece of technical note. The processing of the data seems really fast. My major concern is whether the authors could expand the species in which this pipeline can be applied to. SL trans-splicing is observed in many nematode species, and RNA-seq data are available in modENCODE. Comparative genomics analyses have been performed among these species (e.g. PMID: 22772596, 18218978). If this pipeline can be applied to other nematode species (especially C. briggsae, C. remanei, and C. brenneri, it would have a much greater impact.

>>> SL-quant can indeed be applied to species beyond C. elegans. It only requires (1) the genome sequence, (2) the SL sequences and (3) the gene annotation of the species. As a proof of concept, we have applied SL-quant to datasets from C. briggsae (the datasets from PMID 18218978), C. remanei (modENCODE_4206), C. brenneri (modENCODE_4705), and from the non-nematode T. brucei (SRR038724). As we could not find annotation for the SL sequences of C. briggsae and C. remanei, we used the set of consensus SL sequences for nematodes identified in (PMID 24130571) instead. The results of the SL trans-splicing quantification are summarized in a new table 3. Hundreds of thousands of trans-splicing events were identified and most of them originate from the expected consensus acceptor sites. This demonstrates that SL-quant can be applied to various species in the nematode phylum and beyond.

-A minor comment: The distribution of length in Figure 2C is a bit surprising. Can this be an artifact of the pipeline? I cannot think of any, but the authors may want to be more careful about it.

>>> The length of reads alignment to the SL-sequence depends on:
-Where the reverse transcription stops during the first strand synthesis of the library preparation. Does it reach the 5' end of the RNA fragment or is it blocked prematurely because of the cap or secondary structures?
-Where the second strand synthesis starts. In common RNA-seq library preparation, the reaction is primed by RNA oligonucleotides generated by the digestion of the RNA-DNA duplex obtained after the first strand-synthesis. Therefore, the 5' end of the final dsDNA is truncated.
-The minimal length for an alignment to be reported as significant by BLAST.
We assume that we are not finding many SL-sequences longer than 11 nt in the reads as a result of the first two points indicated above, while 9 nt appears to be the minimal length for an alignment to be significant in BLAST (with our settings, which are detailed in the method section). This issue is now more carefully explained in the manuscript. To further investigate this issue, we now applied SL-quant to an additional C. elegans dataset (SRR2832497) generated using an alternative library preparation protocol based on the direct ligation of adapters on the RNA, which ensures that the full RNA fragments are sequenced. Consistent with our interpretation, the distribution of read alignment length from this dataset ranges from 9 to 22 using the same BLAST settings (see the attached supplementary_figure_for_referees.pdf file, panel A).

-Do other methods have a similar results?

>>> Using the re-implementation of the method used in Tourasse et al., the minimal alignment length drops to 6 nt – as expected given the cutadapt parameters – but the distribution still peaks at 10-11 nt with almost no longer alignments (see the attached supplementary_figure_for_referees.pdf file, panel B). This is consistent with the idea that the length distribution is dependent on the data (presumably because of the library preparation), and not on the method.

Reviewer #2: Major comments:

-Paper derives the conclusion purely based on 2 samples. This is not enough to show the performance of the introduced method. Authors are encouraged to use simulated data and study the effect of read length, throughput and error rate on the accuracy of the proposed method.

>>> As stated in the response to the editor and reviewer 1, we agree that two samples

is not enough. We have therefore analysed six additional samples from various organisms. Library preparation protocols, read length and throughput vary greatly in those datasets. This shows – perhaps more convincingly than it would with simulated reads – that SL-quant can be applied to a broad range of data. The results of the SL trans-splicing quantifications are summarized in the new table 3

-For the reader from the human genomics background it will be helpful if the authors provide the analogy with human trans splicing and fusion events, which are usually detected from RNA-Seq data. Explaining how the short spliced leader (SL) are related to human trans splicing and fusion events can be helpful. Authors can refer to this recent paper : Mangul, Serghei, et al. "ROP: Dumpster Diving in RNA-sequencing to find the source of 1 trillion reads across diverse adult human tissues." Genome biology 19.1 (2018): 36.

>>> We now mention in the manuscript that trans-splicing exists in human as well, although it happens at a low frequency.

-Proposed method can work on any BAM file with both mapped and unmapped reads. This is FANTASTIC, and I am so glad authors are aware of importance of re-suing previously generated data. Is there a minimum read length, which method can handle? For example, will 50 bp be enough?

>>> We thank the referee for his/her enthusiasm. There is no real minimal read length for the method to work, but ideally, the read should be sufficiently long as to allow them to map uniquely after trimming of the SL-sequence. The number of nucleotides trimmed depends on the dataset (organism, library preparation, …) and can vary from read to read. Nevertheless, 50 nt reads are certainly enough in C. elegans. Indeed, we successfully applied SL-quant to the 41 nt (trimmed down to 19-32 nt) reads of the SRR2832497 dataset. The read length in the various datasets tested is now clearly stated in table 3.

-It is great to see that the proposed method can take the pairing information into account. Authors are recommended to comments how the proposed strategy compares to Nicolae, Marius, et al. "Estimation of alternative splicing isoform frequencies from RNA-Seq data." Algorithms for molecular biology 6.1 (2011): 9 in terms of modeling the fragment length distribution.

>>> The fragment length distribution is not directly modelled by SL-quant. In fact, pairing information is used at only two steps in paired-end mode: when the reads are being pre-filtered (fragment length is irrelevant here since only one mate is mapped) and during the remapping of the SL-containing reads by HISAT2. For this last step, we kept the default HISAT2 parameters regarding the maximum fragment length allowed for concordant mapping (ignoring introns), which is 500 nt.

-Section SL_containing reads identification. More details about the real data are needed. For example, how library were prepared? Number of mapped and unmapped reads? Read length?

>>> These are indeed important information that are now detailed in the methods section of the manuscript and table 3. The number of mapped reads is not explicitly indicated but can be inferred from the total number of reads and the number of input reads (= the number of unmapped reads in single-end mode).

-Effect of library preparation protocol needs to be discussed? For example polyA vs ribo depletion

>>> As stated above in the response to reviewer 1 (minor comment), the library preparation (random priming vs ligation method) is of great importance as it affects whether the 5' end of the SL-sequences can be included in the reads. This is now discussed more carefully in the "SL-containing reads identification" section. Concerning poly-A versus ribodepletion, we do not expect a dramatic impact because both protocols enrich for mRNA and SL-containing reads. To make sure of this, one should compare libraries made with either poly-A selection or ribodepletion from exactly the same RNA samples. To the best of our knowledge, such datasets are not available in

C. elegans.

-Paper discuss the detection power of methods. It would be helpful if authors can define standard statistical measures. Such as PPV, Sensitivity and explain how they define TP, FP, FN

>>> Sensitivity, specificity, TP and FP are now clearly defined in the relevant sections of the manuscript (see also the point below). We did not report PPV and therefore did not define it.

-It is not clear how Specificity is defined? To define Specificity one needs to define TN. It is not clear how TN are defined in this context

>>> In the context of the prediction of gene position in operon based on SL-quant results, the definition of TN is straightforward: it is a gene correctly called as not being in positions two and over within operons by our predictor. Specificity, TPR and FPR can therefore be calculated exactly as shown in the ROC curve (figure 4.B). However, when comparing the methods on their ability to call trans-splice sites, the concept of sensitivity becomes relative and cannot be precisely calculated as the truth is unknown. However, we used the following approximations: (1) Sensitivity (TN/N) can be expressed as [1-(FP/N)] because TN=(N-FP); (2) N (the number of negative calls) is approximated as constant because regardless of the method used and dataset studied, ~99.99% of the genome is called negative for trans-splice sites; (3) FP (the number of false positives) is approximated as genomic positions called trans-splice sites not bearing consensus sequences. According to those reasonable approximations, we consider a method “more specific” if it calls less non-consensus trans-splice sites than another method. The reviewer was right to point out this important issue, which is now more carefully explained in the manuscript.

-The authors have done great job documenting the method. However, it would be really helpful to provide full installation scripts. Which will provide one line solution to install all the dependencies. Right now the commands are provided for Mac only. The installation script can be based on conda install, pip or brew.

>>> Actually, the commands were provided for linux as well, based on linuxbrew, the linux equivalent of brew. While the installation is not a one-liner, we believe that it is sufficiently straightforward as to allow seamless installations of SL-quant for most users. Compiling the installation lines in one installation script can perhaps be dangerous if the user does not want to install some dependencies (for instance if he already has a brew-independent installation, this can lead to conflicts). We consider the current step-by-step approach of the installation safer for now. Nevertheless, we will keep the reviewer recommendation in mind for future development of the method and documentation.

Minor comments:
-Usually the blast is referred with all capitals i.e. BLAST. Also it needs to be clarified , if the original BLAST was used or megablast by BLAST+

>>> We used the BLAST+ suite that allows selection from a range of ‘tasks’ (megablast (default) ,blastn, blastn-short, …). Within SL-quant, the task ‘blastn’ is used. This has been clarified in the method section. BLAST is now written in capitals.

Reviewer #3:
The authors describe a method for analyzing unmapped RNA-seq reads to find signatures for trans-splicing. Reads are BLAST-aligned to the SL sequence and, where it is detected, it is trimmed off and the reads are realigned with HISAT2 and genes are quantified with featureCounts.

Results are validated by considering factors such as how specific the SL mappings are to the 3' end of the reads, whether expected motifs are present, and whether SL2 events seem specific to genes beyond the first in the operon.

The authors argue, and it is true as far as I could find, that this is the first software tool for automatically detecting trans-splicing from RNA-seq reads.

Powered by Editorial Manager® and ProduXion Manager® from Aries Systems Corporation

The software is well documented and is distributed under the MIT license.

Major comments:

-The authors argue that the tool is fast since it completes in 10 minutes. It would be helpful for the reader if this could instead be expressed as the number of reads (with unpaired and paired considered separately) processed per unit time. Otherwise it is hard to extrapolate from the 10-minute result. The number of reads in the two evaluated datasets should be reported.

>>> The number of input reads – the reads that are effectively blasted – is indicated in table 1 and the new table 3. About 106 reads are processed within five minutes. As this is an important point, we now indicate this information in the main text as well. Note that the speed per input read is not so great (the bottleneck being the BLAST step), but one of the major features of SL-quant that make it efficient is its pre-filtering step: only the reads likely to carry an SL sequence are taken as input by BLAST. The total number of reads in the evaluated datasets, which is much larger, is now also reported in the tables 1 and 3.

Minor comments:

-"Every run 5 completed within 10 min" -- the authors should clarify how many simultaneous threads were used. Based on the parameters listed in the Methods section, I think this result is using a single thread.

>>> We used the default options of SL-quant, which is 4 threads as detailed in the “advanced usage” section of our github page. This option can be modified according to the instruction on our github page (‘advanced usage’ section). We agree that is an important information and it has been clarified in the manuscript.

-"8 GO RAM" should presumably be "8 GB RAM"

>>>This was corrected accordingly.

Additional Information:

**Question**	**Response**
Are you submitting this manuscript to a special series or article collection?	No
**Experimental design and statistics**<br><br>Full details of the experimental design and statistical methods used should be given in the Methods section, as detailed in our Minimum Standards Reporting Checklist. Information essential to interpreting the data presented should be made available in the figure legends.<br><br>Have you included all the information requested in your manuscript?	Yes
**Resources**	Yes

Powered by Editorial Manager® and ProduXion Manager® from Aries Systems Corporation

A description of all resources used, including antibodies, cell lines, animals and software tools, with enough information to allow them to be uniquely identified, should be included in the Methods section. Authors are strongly encouraged to cite Research Resource Identifiers (RRIDs) for antibodies, model organisms and tools, where possible.<br><br>Have you included the information requested as detailed in our Minimum Standards Reporting Checklist?	
**Availability of data and materials**<br><br>All datasets and code on which the conclusions of the paper rely must be either included in your submission or deposited in publicly available repositories (where available and ethically appropriate), referencing such data using a unique identifier in the references and in the “Availability of Data and Materials” section of your manuscript.<br><br>Have you have met the above requirement as detailed in our Minimum Standards Reporting Checklist?	Yes

Powered by Editorial Manager® and ProduXion Manager® from Aries Systems Corporation

Manuscript with DOI
Click here to view linked References
Click here to access/download;Manuscript;20180601_SL-quant_tracked with DOI.docx

SL-quant: A fast and flexible pipeline to quantify spliced leader trans-splicing events from RNA-seq data.

Carlo Yague-Sanz (carlo.yaguesanz@unamur.be)[1*] and Damien Hermand (damien.hermand@unamur.be)[1]

[1] URPhyM-GEMO, University of Namur, 5000 Namur, Belgium.

[*] To whom correspondence should be addressed

Corresponding author ORCID: 0000-0002-9941-9703

Running head: Quantification of trans-splicing events from RNA-seq data

ABSTRACT

Background

The spliceosomal transfer of a short spliced leader (SL) RNA to an independent pre-mRNA molecule is called SL trans-splicing and is widespread in the nematode *C. elegans*. While RNA-seq data contain information on such events, properly documented methods to extract them are lacking.

Findings

To address this, we developed *SL-quant,* a fast and flexible pipeline that adapts to paired-end and single-end RNA-seq data and accurately quantifies SL trans-splicing events. It is designed to work downstream of read mapping and uses the reads left unmapped as primary input. Briefly, the SL-sequences are identified with high specificity and are trimmed from the input reads, which are then re-mapped on the reference genome and quantified at the nucleotide position level (SL trans-splice sites) or at the gene level.

Conclusions

SL-quant completes within 10 minutes on a basic desktop computer for typical *C.elegans* RNA-seq datasets, and can be applied to other species as well. Validating the method, the SL trans-splice sites identified display the expected consensus sequence and the results of the gene-level quantification are predictive of the gene position within operons. We also compared *SL-quant* to a recently published SL-containing read identification strategy which revealed being more sensitive, but less specific than *SL-quant*. Both methods are implemented as a bash script available under the MIT licence at https://github.com/cyaguesa/SL-quant. Full instructions for its installation, usage, and adaptation to other organisms are provided.

KEYWORDS: NGS, RNA-seq, maturation, trans-splicing, sequence analysis.

FINDINGS

Background

The capping, splicing and polyadenylation of eukaryotic pre-mRNAs are well-studied maturation processes that are essential for proper gene expression in eukaryotes [1]. Much less is known about spliced leader trans-splicing, a process by which a capped small nuclear RNAs called spliced leader (SL) is spliced onto the 5'-end of a pre-mRNA molecule, substituting for canonical capping [2] (**fig 1.A)**. SL trans-splicing has a patchy phylogenetic distribution ranging from protists [3] to bilaterian metazoans, including nematodes, rotifers [4] and even chordates [5]. It appears not conserved in mammals, although 'non-SL' trans-splicing events – when exons from two different RNA transcripts are spliced together – have been detected at low frequency [6]. In contrast, SL trans-splicing is widespread in the *C. elegans* nematode where there are two classes of SL, called SL1 and SL2, which trans-splice about 70% of the mRNA transcripts. Strikingly, the SL2 trans-splicing is highly specific for genes in position two and over within operons that range from two to eight genes expressed from a single promoter [7].

While the function of SL trans-splicing begins to be elucidated [8], its regulation remains unclear. To study this question, two main strategies have been proposed to exploit RNA-seq data in order to quantify SL trans-splicing. The first one involves the mapping of the reads to a complex database containing all the possible trans-spliced gene models [9, 10]. The creation of such a database requires the *in silico* trans-splicing of every SL sequence isoform (12 in *C. elegans*) to all the putative trans-splice sites predicted for a gene. In contrast, the second strategy does not rely on trans-splice site annotation or prediction. Instead, the SL

sequences are directly identified in reads partially mapped to the genome or transcriptome [11-13]. However, no implementation of these methods is directly available, which prompted us to develop, test and optimize *SL-quant*, a ready-to-use pipeline that applies the second strategy to rapidly quantify SL trans-splicing events from RNA-seq data.

Pipeline overview

In order to search for SL sequences in a limited number of reads, only unmapped reads are used as input for *SL-quant*, assuming that reads containing the SL-sequence (or the 3' end of it) would not map on the reference genome or transcriptome (**figure 1.B**). This implies that a first round of mapping must precede the use of *SL-quant*. It must be performed end-to-end in order to guarantee that reads originating from trans-spliced RNA fragments do not map. Beside this specification, any bam file containing unmapped reads can be fed into *SL-quant*, making it particularly well suited for subsequent analyses of previously generated data.

In the case paired-end reads are available, only the unmapped reads originating from the left-most ends of the fragments are considered. In addition, we developed an optimized paired-end mode (*-p --paired* option) that further limits the search for SL-containing reads by filtering out the unmapped reads whose mates are also unmapped. This assumes that only the left-most read of a pair originating from a trans-spliced fragment would not map due to the SL-sequence while the other one would map (**figure 1.C**). It is generally true unless the fragment is so small that the mates significantly overlap with each other (**figure 1.D**).

To identify SL trans-splicing events, the input reads are aligned locally to the SL sequences with BLAST [14]. Reads whose 5' end belongs to a significant alignment (e-value < 5%) that covers the 3' end of the SL-sequence (**figure 2.A**, left panel) are considered *SL-*

containing reads. Then, the SL-containing reads are trimmed of the SL-sequence (based on the length of the BLAST alignment) and mapped back on *C. elegans* genome with HISAT2 [15]. Finally, the re-mapped reads are counted at the gene level with *featureCounts* [16] to obtain a quantification of the SL1 and SL2 trans-splicing events per genes.

SL-containing reads identification

We tested *SL-quant* on the single-end modENCODE_4594 [17] dataset (2.5×10^6 unmapped reads) and the paired-end SRR1585277 [18] dataset (1.3×10^6 unmapped left reads) using a desktop computer with basic specifications. Every run completed within 10 min using 4 threads, with a processing rate of about 10^6 unmapped reads by 5 minutes.

In order to assess the specificity of the BLAST alignments, we reasoned that reads originating from a trans-spliced RNA would align to the 3' end of the SL sequence from their 5' end, while random alignment would start anywhere (**figure 2.A**). The fact that 94% of significant alignments were in that specific configuration indicates good specificity (**table 1** and **figure 2.B**). In contrast, we obtained less than 0.3% with randomly generated reads. In paired-end mode, less alignments were found but a slightly higher proportion of them (95%) were in proper configuration and considered SL-containing reads. This was expected given the more stringent pre-filtering implemented in that mode. When considering only the non-significant alignments, we obtained intermediate proportions of proper configuration (15-20%), suggesting that most, but not all, of those non-significant alignments were spurious.

Despite the *C. elegans* SL sequences being 22 nucleotides (nt) long, most alignments cover them on only 10-11 nt **(figure 2.C)**, with a preference for 10 nt alignment for SL1-containg reads and 11 nt alignments for SL2-containing reads. This could be caused by reverse

transcriptase drop-off during the library preparation due to secondary structure and the proximity of the hyper-methylated cap at the 5' end of the SL. Moreover, in classical RNA-seq library preparation protocols, the second-strand synthesis is primed by RNA oligonucleotides generated by the digestion of the RNA-DNA duplex obtained after the first strand synthesis. This results in truncated dsDNA fragments that do not preserve the 5' end of the original RNA fragments [19].

SL trans-splice sites identification.

While we designed SL-quant with the idea of quantifying SL trans-splicing events by gene, it is also possible to use it to identify the 3' trans-splice sites at single nucleotide resolution. SL trans-splice sites are known to display the same UUUCAG consensus as *cis*-splice sites [20], which could be verified with our method (**figure 3.A,B**). Previous work described a significant switch from A to G after to consensus sequence (position +1) for the SL1 trans-splice sites compared to SL2 trans-splice site [20]. At that position, we observed a decreased preference for A for the SL1 trans-splice sites, but no significant enrichment in G. This discrepancy could be due to the fact that we identified (and included in the consensus) about 20 times more SL1 trans-splice sites than previously reported.

As SL trans-splice sites (and splice sites in general) contain an almost invariant AG sequence, we reasoned that non-AG splice sites were potential *'spurious'* trans-splice sites. In order to assess the performances of our method, we considered identified sites bearing the 'AG' consensus as true positives (TP). Reciprocally, we considered any other sites identified as false positives (FP) although we cannot completely exclude the existence of non-consensus splice sites. These reasonable approximations allow us to characterize our method despite not

knowing the ground truth. Indicating excellent specificity (ability to exclude FP), 98% of the sites identified by *SL-quant* display the AG consensus, regardless of the mode used (single or paired) and the dataset studied (**table 2**).

Comparison with a previous method

We also compared our method with a re-implementation of the SL-containing read identification strategy previously reported [13]. Briefly, the unmapped reads whose 5' end align to the SL sequences (or their reverse complement) on at least 5 nt with at most 10% mismatch are considered SL-containing reads. The alignment is realized with *cutadapt* [21] that directly trim the SL-sequences from the unmapped reads so they can be re-mapped to the genome.

Compared to *SL-quant*, this conceptually similar method was faster and identified almost twice the number of SL-containing reads from the real datasets, and 150 times the number of SL-containing reads from random reads (**table 2**). More splice-sites were identified, but the proportion of spurious (non-consensus) trans-splice sites increased almost 5-fold (**figure 3.C**).

All in all, the method developed in [13] has a higher detection power but appears less specific than *SL-quant*. Nevertheless, we consider it an interesting option for applications requiring more sensitivity (ability to detect TP) than specificity. Therefore, we decided to re-implement it within *SL-quant* as an *[-s --sensitive]* option with the following enhancement:

- The input reads, if strand-specific, are aligned to the SL sequences only (not their reverse complement).
- With paired-end data in single-end mode, only the left-most unmapped reads are considered as input.

- With paired-end data in paired-end mode, only the left-most unmapped reads whose mates are mapped are considered as input.

These modifications significantly improved the specificity of the method (although not to the level of *SL-quant*) with almost no compromise on sensitivity regarding SL trans-splice sites detection (**figure 3.C**) or SL-containing reads identification (**table 2**).

Gene level quantification

Finally, we tested *SL-quant* for its ability to predict gene position within operons as SL2-trans-splicing is the best predictor of transcription initiated upstream of another gene [10] **(figure 4.A).** Using the ratio of *SL2/(SL1+SL2)* from the *SL-quant* output as a predictor of gene positions in operons, ROC curve analysis reveals high true positive rate (>90%) at a 5% false discovery rate threshold, regardless of *SL-quant* options **(figure 4.B)**. However, when tolerating more false positives, *SL-quant* in *sensitive* mode is a superior predictor.

Conclusion

To sum up, *SL-quant* is able to rapidly and accurately quantify trans-splicing events from RNA-seq data. It comes as a well-documented and ready-to-use pipeline in which two main options were implemented to fit the type of input data and the intended usage of the quantification (**figure 5**). Importantly, this work provides means to test and validates SL trans-splicing quantification methods that might serve as a baseline for future development of such methods.

Recently, the hypothesis that the SL trans-splicing mechanism originates from the last eukaryotic common ancestor has been proposed to explain its broad phylogenetic distribution

[22]. Given the number of concerned species, the continuously decreasing cost of RNA-seq experiments and the thinner line between model and non-model organisms, it is likely that the SL trans-splicing will be studied in a growing number of species. Therefore, a procedure to adapt *SL-quant* to species beyond *C. elegans*, requiring only a few steps, is detailed online. As a proof of concept, we successfully applied *SL-quant* to six additional RNA-seq libraries from five different species (table 3). In the near future, we anticipate that the application of *SL-quant* to various datasets might become instrumental in unveiling trans-splicing regulation in the model organism *C. elegans* and beyond.

METHODS

We ran *SL-quant* with 4 threads (default) on the modENCODE_4594, modENCODE_4705, modENCODE_4206 [17], SRR2832497 [23], SRR440441, SRR440557 [24], SRR038724 [25] and SRR1585277 [18] poly-A + datasets using a desktop computer with a 2.8 GHz processor and 8 GB RAM. The *C. elegans, C. briggsae, C. brenneri and C. remanei* reference genome and annotation (WS262) were downloaded from wormbase [26]. The *T. brucei* reference genome and annotation (Apr_2005 version) were downloaded from Ensembl [27]. The read mapping steps prior to using *SL-quant* and at the end of the pipeline were performed using *HISAT2* [15] (v 2.0.5) with parameters --no-softclip --no-discordant --min-intronlen 20 --max-intronlen 5000. As we noticed adaptor contamination in the modENCODE_4594 dataset, *trimmomatic* [28] (v 0.36) was used to trim them off prior to the mapping. *Samtools* [29] (v 1.5), *picard* [30] (v 2.9) and *bedtools* [31] (v 2.26) were used to convert and/or filter the reads at various stages of the pipeline. BLAST+ (v 2.6) [14] was used to align the reads locally to the relevant SL sequences [32, 33] with parameter -task blastn -word_size 8 max_target_seqs 1. Alternatively, *cutadapt* (v 1.14) [21] was used to directly trim the SL sequences from the reads with parameters -O 5 -m 15 --discard-untrimmed.

FeatureCounts [16] was used to summarize re-mapped SL-containing reads at the gene level. *Bedtools* [31] was used to summarize mapped SL-containing reads at the genomic position level and to generate random reads by randomly sampling the *C. elegans* genome for 50 nt segments. Sequence logo were made with *weblogo* [34]. Finally, R [35] (v 3.4) was used for analysing and visualizing the data.

Availability of supporting source code and requirements

Project name: SL-quant

Project home page: https://github.com/cyaguesa/SL-quant

Operating system(s): UNIX-based systems (tested on macOS 10.12.6, macOS 10.11.6, Ubuntu 14.04)

Programming language: Shell, R.

Other requirements: The BLAST+ suite (2.6.0 or higher), samtools (1.5 or higher), picard-tools (2.9.0 or higher), featureCounts from the subread package. (1.5.0 or higher), bedtools (2.26.0 or higher), cutadapt (1.14 or higher), hisat2 (2.0.5 or higher). Installation instruction for those requirements is provided online.

License: MIT

RRID: SCR_016205

Availability of supporting data

The data sets supporting the results of this article are available in the modMine or the European Nucleotide Archive (ebi-ENA) repositories, under the identifiers modENCODE_4594, modENCODE_4705, modENCODE_4206, SRR1585277, SRR2832497, SRR440441, SRR440557, SRR038724. Snapshots of the code and other supporting data are available in the GigaScience repository, GigaDB [36].

DECLARATIONS

List of abbreviations:

NS: non-significant; nt: nucleotide; RNA: ribonucleic acid; ROC: receiver operatic characteristic; SL: spliced leader

Ethics approval and consent to participate: **NA**

Competing interests: The authors declare that they have no competing interests.

Consent for publication: **NA**

Funding: This work was supported by the FNRS-FRIA.

Authors' contributions: C.Y. designed, implemented and tested the pipeline. D.H. and C.Y. wrote the manuscript. D.H. supervised the project.

Acknowledgements: We thank Olivier Finet and Fanélie Bauer for critical reading of the manuscript.

TABLES

				significant alignments		NS alignments	
dataset	method	total reads	input reads	total	properly configured	total	properly configured
SRR1585277	SL-quant	40x10^6	1.3x10^6	71512	67021 (94%)	70211	10359 (15%)
	SL-quant -p	40x10^6	0.9x10^6	67463	64010 (95%)	47596	9849 (21%)
modENCODE_4594	SL-quant	30x10^6	2.5x10^6	168351	158529 (94%)	100139	20417 (20%)
random	SL-quant	1x10^6	1.0x10^6	12788	36 (0.3%)	43501	83 (0.2%)

Table 1. Identification of SL-containing reads by *SL-quant*. SL-containing reads are defined as reads with significant and properly configured alignment to the SL sequences (6th column). NS: non-significant.

dataset	method	run time	mapped SL-containing reads	trans-splice sites	site is "AG" consensus (%)
SRR1585277	SL-quant	4m02s	65126	6301	6149 (98%)
	SL-quant -p	5m14s	61451	6539	6402 (98%)
	SL-quant -s	2m45s	120542	8770	8254 (94%)
	SL-quant -s -p	6m58s	114948	8436	7957 (94%)
	Tourasse	4m45s	120710	8932	8260 (92%)
modENCODE_4594	SL-quant	9m51s	146358	8247	8081 (98%)
	SL-quant -s	3m10s	258706	10735	9948 (93%)
	Tourasse	5m08s	259284	11155	9953 (89%)
random	SL-quant	3m20s	53	52	34 (65%)
	SL-quant -s	1m23s	5757	5692	5612 (99% [a])
	Tourasse	2m24s	8890	8777	5612 (64%)

Table 2. Performances of *SL-quant* with various parameters. -p: paired-end mode. -s: sensitive mode.

organism	dataset	read length	total reads	input reads	mapped SL-containing reads	trans-splice sites (% AG)
C.elegans	SRR1585277	76 nt	40x10^6	1.3x10^6	120542	8770 (94%)
	modENCODE_4594	76 nt	30x10^6	2.5x10^6	258706	10735 (93%)
	SRR2832497 (*)	41 nt	4x10^6	1.8x10^6	16307	4882 (87%)
C. briggsae	SRR440441	42 nt	11x10^6	5.7x10^6	117738	8382 (93%)
	SRR440557	42 nt	12x10^6	4.8x10^6	176205	11495 (92%)
C. brenneri	modENCODE_4705	76 nt	4x10^6	0.4x10^6	74689	8891 (97%)
C. remanei	modENCODE_4206	76 nt	9x10^6	1.8x10^6	248335	11223 (92%)
T. brucei	SRR038724	35 nt	8x10^6	2.2x10^6	40320	6703 (89%)

Table 3. *SL-quant* can be applied to a wide range of datasets from various species, with varying read length and made with various library preparation protocols. The datasets modENCODE_4594, SRR2832497 and SRR038724 are single-end, the others are paired. The asterisks (*) for the SRR2832497 denotes that the second-strand synthesis was made using a ligation-based protocol instead of the classical random priming protocol. All datasets were analysed with the same *SL-quant* parameters: single-end mode with the -s --sensitive option.

ENDNOTES

[a] The very high proportion of "AG" site for the random dataset is an artefact caused by the fact that the reads were generated from randomly sampling the genome and that all the *C. elegans* SL sequences end by AG.

FIGURE LEGENDS

Fig.1. Trans-splicing and RNA-seq. A) The trans-splicing process. Splice leader RNA precursors (SL RNA) are small nuclear RNAs capped with a trimethyl-guanosine (TMG). The 5'-region of the SL RNA including the TMG cap, is spliced on the first exon of the pre-mRNAs. **B)** Reads originating from trans-spliced RNA fragments do not map end-to-end to the reference genome. **C)** The left-most reads (R2) of a read pair does not map end-to-end to the reference. **D)** Special case when the paired-end reads "dovetail" and both reads do not map end-to-end to the reference due to the SL sequence.

Fig.2. Configuration of the BLAST alignments. A) *In SL-quant*, the BLAST alignments are considered as properly configured if starting from the 5'end of the unmapped read and ending at the 3' end of the SL sequence. **B)** Proportion of properly configured alignments out of the

significant alignment identified by *SL-quant* in single and paired-end (-p) mode on the *SRR1585277* dataset, or on 10^6 random reads in single-end mode. **C)** Number of properly configured significant alignments found by *SL-quant* on the *SRR1585277* dataset (single-end mode) by alignment length on the SL1 or SL2 sequences.

Fig.3. SL-sites consensus sequence. A) Sequence logo of the sequence environment surrounding SL1 or **B)** SL2 trans-splice sites determined by *SL-quant* on the *SRR1585277* dataset in single-end mode. **C)** Proportion of AG sequences in SL trans-splice sites identified by SL-quant on the *SRR1585277* dataset with the method used in *Tourasse et al*, *2017* and with *SL-quant* in single-end mode with or without the sensitive option (-s).

Fig.4. Prediction of genes position in operons. A) Number of SL1 and SL2 trans-splicing events by genes as calculated by *SL-quant*. Genes annotated as downstream in the operons are represented as red dots. **B)** Receiver operating characteristic (ROC) curve analysis using the SL2/(SL1+SL2) ratio as a predictor of downstream position in operons for the 5521 genes with at least one trans-splicing event detected. The number of SL1 and SL2 trans-splicing events by genes was calculated by *SL-quant* in single or paired (-p) mode, with or without the sensitive (-s) option. TPR: true positive rate, FPR: false positive rate.

Fig.5. **Recommendations on *SL-quant* usage.**

[-s --sensitive]: it provides increased detection power at the cost of some specificity and it is significantly faster. It is not recommended for applications that are very sensitive to false positives (e.g. trans-splice sites detection) but is an interesting option otherwise (e.g. gene level quantification of SL trans-splicing events).

[-p --paired]: a more stringent pre-filtering reduces the number of reads aligned to the SL-sequences. It can only be used with paired-end reads. It is not recommended when the average fragment size is small (many “dovetail” reads). It can be used in combination with the [-s –sensitive] option.

REFERENCES

1. Bentley DL. Coupling mRNA processing with transcription in time and space. Nature reviews Genetics. 2014;15 3:163-75. doi:10.1038/nrg3662.
2. Blumenthal T. Trans-splicing and operons in C. elegans. WormBook. 2012:1-11. doi:10.1895/wormbook.1.5.2.
3. Michaeli S. Trans-splicing in trypanosomes: machinery and its impact on the parasite transcriptome. Future Microbiol. 2011;6 4:459-74. doi:10.2217/fmb.11.20.
4. Pouchkina-Stantcheva NN and Tunnacliffe A. Spliced leader RNA-mediated trans-splicing in phylum Rotifera. Mol Biol Evol. 2005;22 6:1482-9. doi:10.1093/molbev/msi139.
5. Vandenberghe AE, Meedel TH and Hastings KE. mRNA 5'-leader trans-splicing in the chordates. Genes & development. 2001;15 3:294-303. doi:10.1101/gad.865401.
6. Mangul S, Yang HT, Strauli N, Gruhl F, Porath HT, Hsieh K, et al. ROP: dumpster diving in RNA-sequencing to find the source of 1 trillion reads across diverse adult human tissues. Genome Biol. 2018;19 1:36. doi:10.1186/s13059-018-1403-7.
7. Blumenthal T, Evans D, Link CD, Guffanti A, Lawson D, Thierry-Mieg J, et al. A global analysis of Caenorhabditis elegans operons. Nature. 2002;417 6891:851-4. doi:10.1038/nature00831.
8. Yang YF, Zhang X, Ma X, Zhao T, Sun Q, Huan Q, et al. Trans-splicing enhances translational efficiency in C. elegans. Genome research. 2017;27 9:1525-35. doi:10.1101/gr.202150.115.
9. Hillier LW, Reinke V, Green P, Hirst M, Marra MA and Waterston RH. Massively parallel sequencing of the polyadenylated transcriptome of C. elegans. Genome research. 2009;19 4:657-66. doi:10.1101/gr.088112.108.
10. Allen MA, Hillier LW, Waterston RH and Blumenthal T. A global analysis of C. elegans trans-splicing. Genome research. 2011;21 2:255-64. doi:10.1101/gr.113811.110.
11. Maxwell CS, Antoshechkin I, Kurhanewicz N, Belsky JA and Baugh LR. Nutritional control of mRNA isoform expression during developmental arrest and recovery in C. elegans. Genome research. 2012;22 10:1920-9. doi:10.1101/gr.133587.111.
12. Boeck ME, Huynh C, Gevirtzman L, Thompson OA, Wang G, Kasper DM, et al. The time-resolved transcriptome of C. elegans. Genome research. 2016;26 10:1441-50. doi:10.1101/gr.202663.115.

13. Tourasse NJ, Millet JRM and Dupuy D. Quantitative RNA-seq meta-analysis of alternative exon usage in C. elegans. Genome research. 2017;27 12:2120-8. doi:10.1101/gr.224626.117.
14. Camacho C, Coulouris G, Avagyan V, Ma N, Papadopoulos J, Bealer K, et al. BLAST+: architecture and applications. BMC Bioinformatics. 2009;10:421. doi:10.1186/1471-2105-10-421.
15. Kim D, Langmead B and Salzberg SL. HISAT: a fast spliced aligner with low memory requirements. Nat Methods. 2015;12 4:357-60. doi:10.1038/nmeth.3317.
16. Liao Y, Smyth GK and Shi W. featureCounts: an efficient general purpose program for assigning sequence reads to genomic features. Bioinformatics. 2014;30 7:923-30. doi:10.1093/bioinformatics/btt656.
17. Gerstein MB, Lu ZJ, Van Nostrand EL, Cheng C, Arshinoff BI, Liu T, et al. Integrative analysis of the Caenorhabditis elegans genome by the modENCODE project. Science. 2010;330 6012:1775-87. doi:10.1126/science.1196914.
18. Kosmaczewski SG, Edwards TJ, Han SM, Eckwahl MJ, Meyer BI, Peach S, et al. The RtcB RNA ligase is an essential component of the metazoan unfolded protein response. EMBO Rep. 2014;15 12:1278-85. doi:10.15252/embr.201439531.
19. Agarwal S, Macfarlan TS, Sartor MA and Iwase S. Sequencing of first-strand cDNA library reveals full-length transcriptomes. Nat Commun. 2015;6:6002. doi:10.1038/ncomms7002.
20. Graber JH, Salisbury J, Hutchins LN and Blumenthal T. C. elegans sequences that control trans-splicing and operon pre-mRNA processing. RNA. 2007;13 9:1409-26. doi:10.1261/rna.596707.
21. Martin M. Cutadapt removes adapter sequences from high-throughput sequencing reads. EMBnet journal. 2011;17 1:pp. 10-2.
22. Krchnakova Z, Krajcovic J and Vesteg M. On the Possibility of an Early Evolutionary Origin for the Spliced Leader Trans-Splicing. J Mol Evol. 2017;85 1-2:37-45. doi:10.1007/s00239-017-9803-y.
23. Ni JZ, Kalinava N, Chen E, Huang A, Trinh T and Gu SG. A transgenerational role of the germline nuclear RNAi pathway in repressing heat stress-induced transcriptional activation in C. elegans. Epigenetics Chromatin. 2016;9:3. doi:10.1186/s13072-016-0052-x.
24. Uyar B, Chu JS, Vergara IA, Chua SY, Jones MR, Wong T, et al. RNA-seq analysis of the C. briggsae transcriptome. Genome research. 2012;22 8:1567-80. doi:10.1101/gr.134601.111.
25. Kolev NG, Franklin JB, Carmi S, Shi H, Michaeli S and Tschudi C. The transcriptome of the human pathogen Trypanosoma brucei at single-nucleotide resolution. PLoS Pathog. 2010;6 9:e1001090. doi:10.1371/journal.ppat.1001090.
26. Stein L, Sternberg P, Durbin R, Thierry-Mieg J and Spieth J. WormBase: network access to the genome and biology of Caenorhabditis elegans. Nucleic acids research. 2001;29 1:82-6.
27. Kersey PJ, Allen JE, Allot A, Barba M, Boddu S, Bolt BJ, et al. Ensembl Genomes 2018: an integrated omics infrastructure for non-vertebrate species. Nucleic acids research. 2018;46 D1:D802-D8. doi:10.1093/nar/gkx1011.
28. Bolger AM, Lohse M and Usadel B. Trimmomatic: a flexible trimmer for Illumina sequence data. Bioinformatics. 2014;30 15:2114-20. doi:10.1093/bioinformatics/btu170.
29. Li H, Handsaker B, Wysoker A, Fennell T, Ruan J, Homer N, et al. The Sequence Alignment/Map format and SAMtools. Bioinformatics. 2009;25 16:2078-9. doi:10.1093/bioinformatics/btp352.

30. Picard tools. http://broadinstitute.github.io/picard.
31. Quinlan AR. BEDTools: The Swiss-Army Tool for Genome Feature Analysis. Curr Protoc Bioinformatics. 2014;47:11 2 1-34. doi:10.1002/0471250953.bi1112s47.
32. Guiliano DB and Blaxter ML. Operon conservation and the evolution of trans-splicing in the phylum Nematoda. PLoS genetics. 2006;2 11:e198. doi:10.1371/journal.pgen.0020198.
33. Bitar M, Boroni M, Macedo AM, Machado CR and Franco GR. The spliced leader trans-splicing mechanism in different organisms: molecular details and possible biological roles. Front Genet. 2013;4:199. doi:10.3389/fgene.2013.00199.
34. Crooks GE, Hon G, Chandonia JM and Brenner SE. WebLogo: a sequence logo generator. Genome research. 2004;14 6:1188-90. doi:10.1101/gr.849004.
35. R Core Team. R: A language and environment for statistical computing. Vienna, Austria: R Foundation for Statistical Computing, 2017.
36. Yague-Sanz C, Hermand D: Supporting data for "SL-quant: A fast and flexible pipeline to quantify spliced leader trans-splicing events from RNA-seq data" GigaScience Database. 2018. http://dx.doi.org/10.5524/100477

Figure 1

Click here to access/download;Figure;fig1_SL-

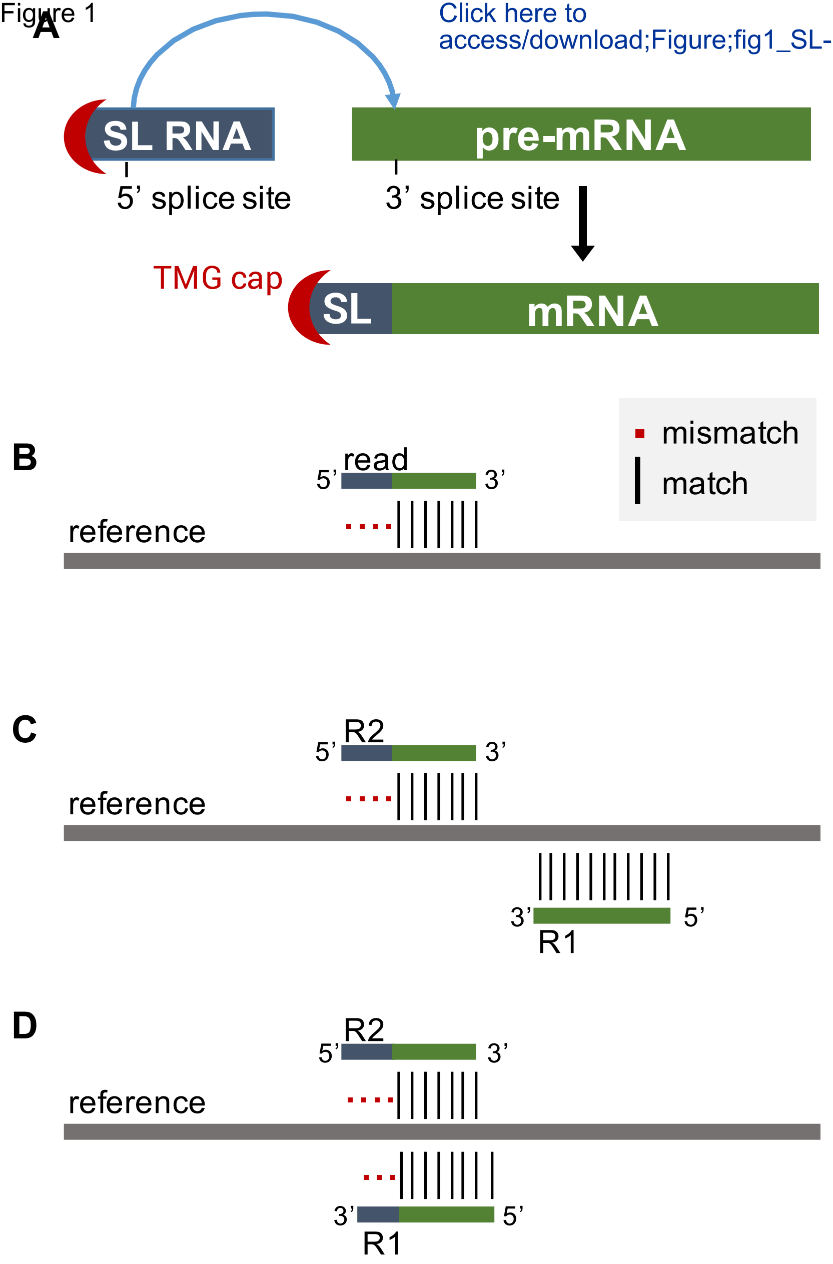

Figure 2

Click here to access/download;Figure;fig2_SL-

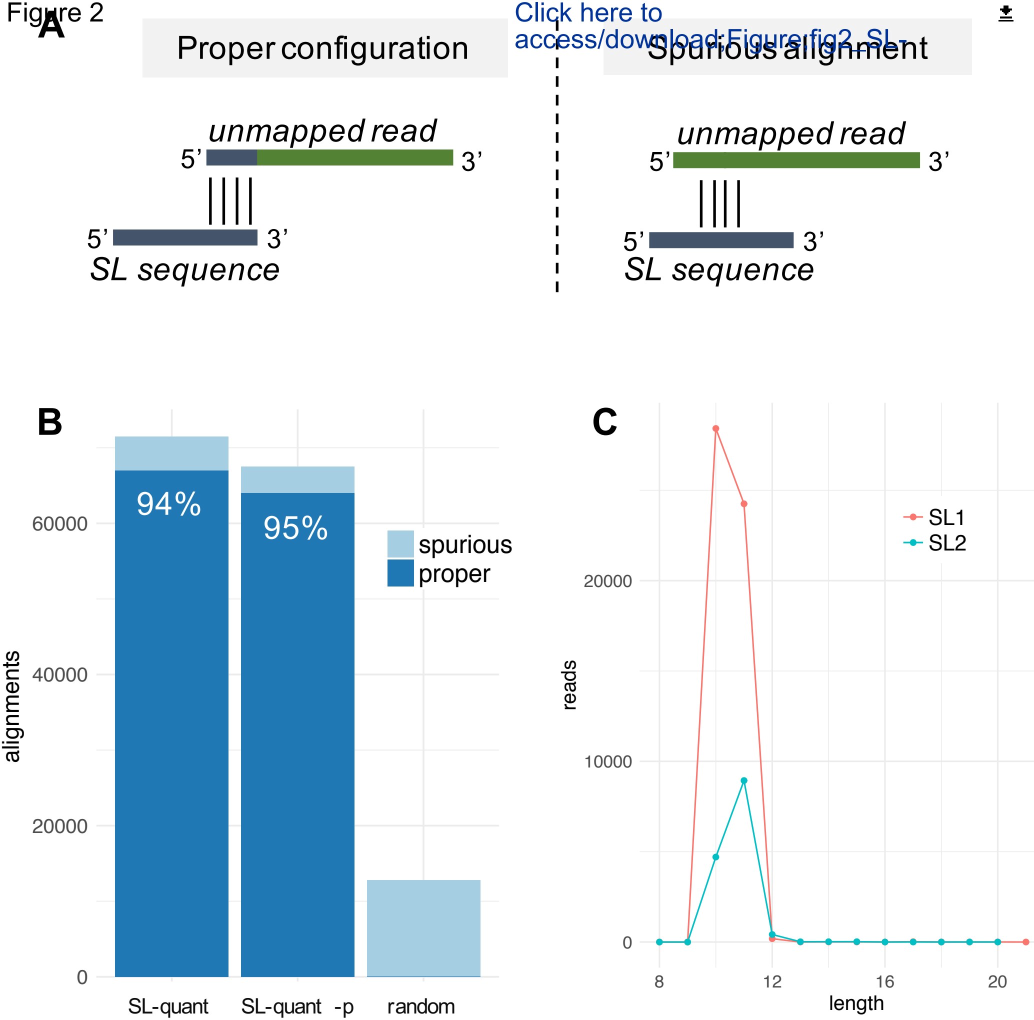

Figure 3

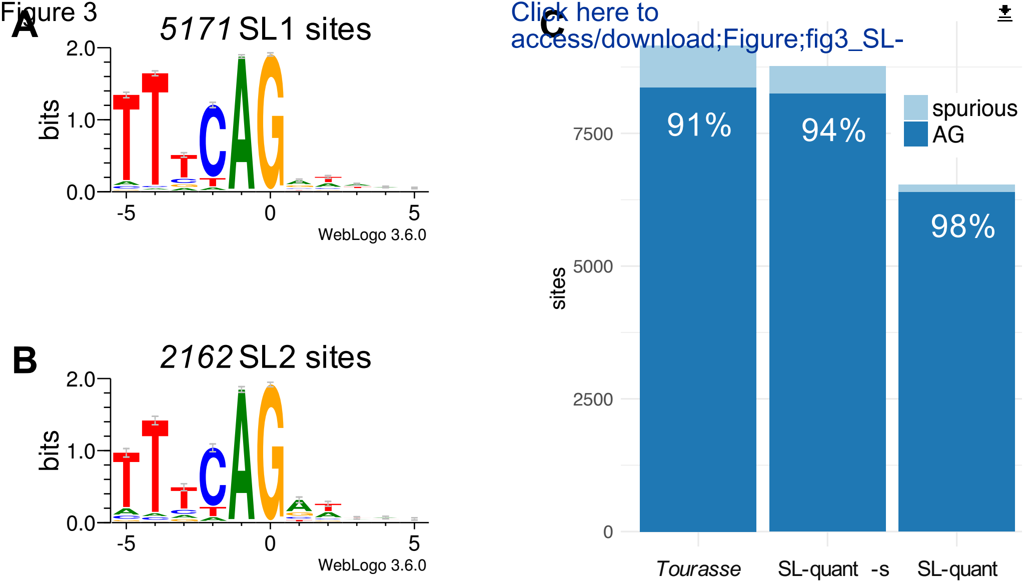

Click here to access/download;Figure;fig3_SL-
A
5171 SL1 sites
2.0
1.0
0.0
bits
-5
0
5
WebLogo 3.6.0
B
2162 SL2 sites
2.0
1.0
0.0
bits
-5
0
5
WebLogo 3.6.0
C
spurious
AG
91%
94%
98%
7500
5000
2500
0
sites
Tourasse
SL-quant -s
SL-quant

Figure 4

Click here to access/download;Figure;fig4_SL-quant.pdf

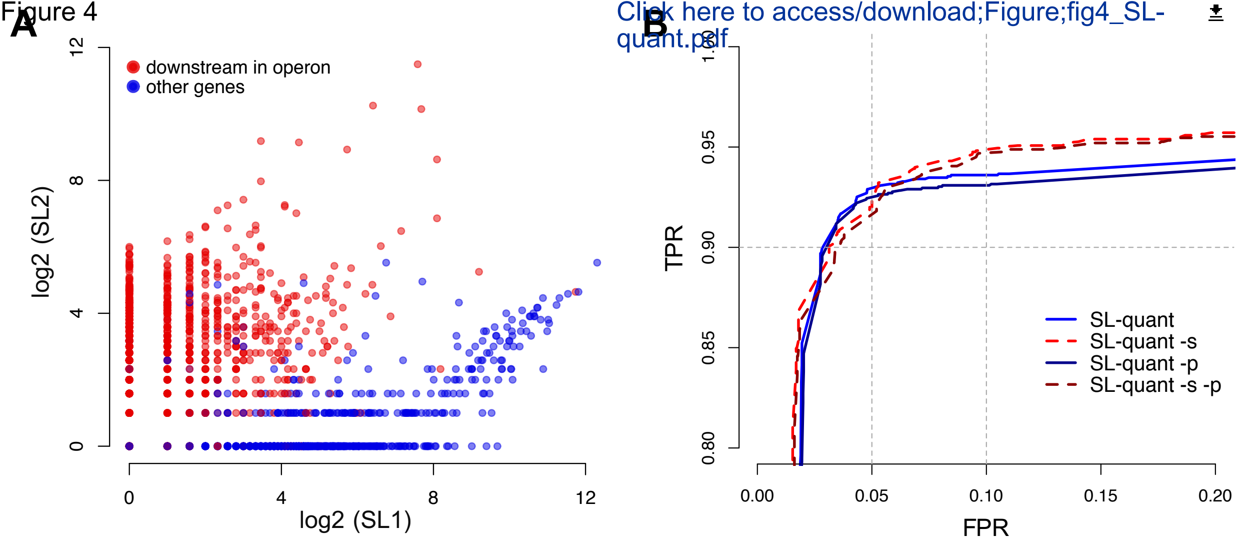

Figure 5

Click here to access/download;Figure;fig5_SL-quant.pdf

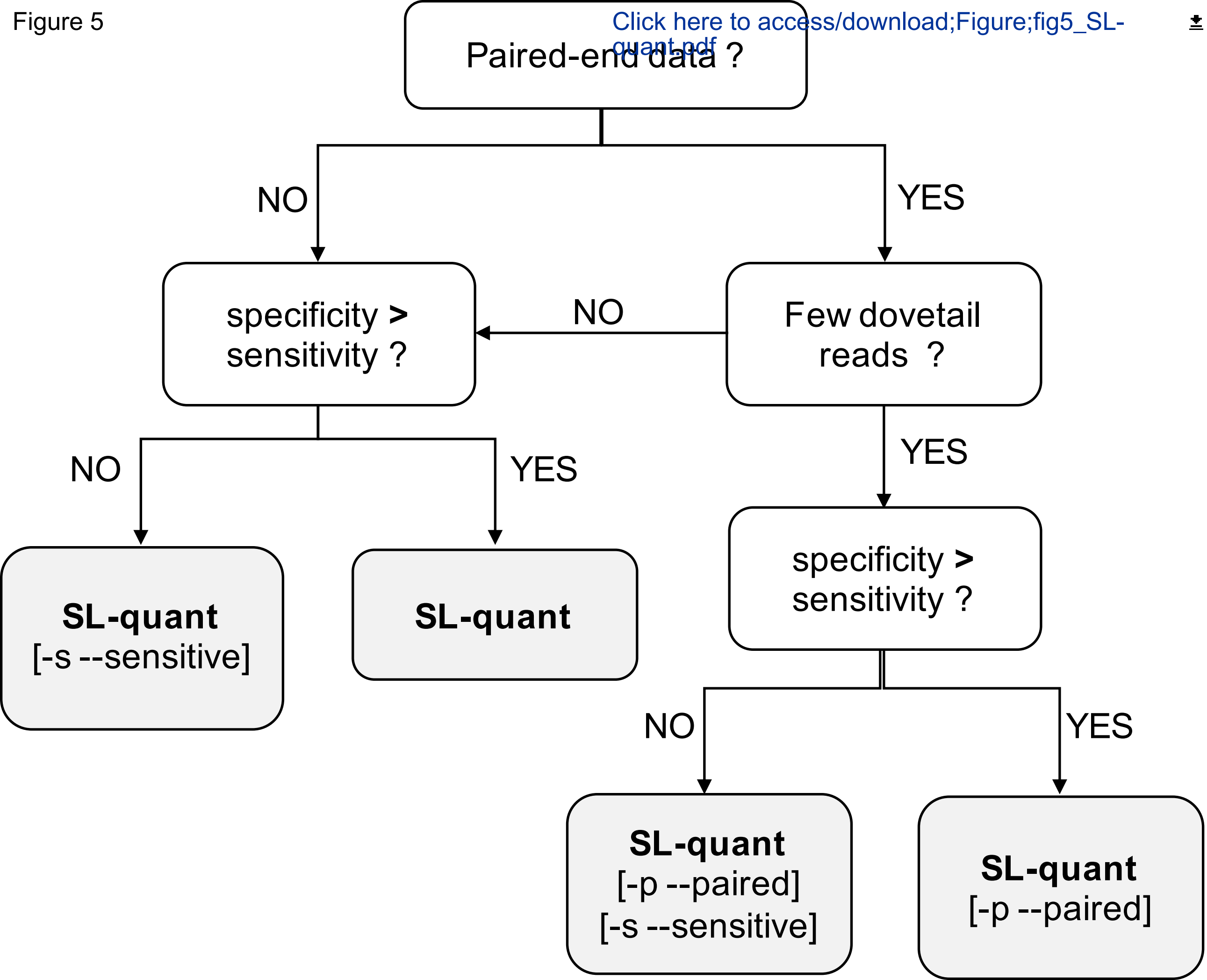

figure in response to reviewers

Click here to access/download
Supplementary Material
supplementary_figures_for_referees.pdf
