# Supplementary material for: SL-quant: a fast and flexible pipeline to quantify spliced leader trans-splicing events from RNA-seq data
Source: Gigascience. 2018 Jul 11;7(7):giy084. doi: 10.1093/gigascience/giy084 (PMC6055573; doi:10.1093/gigascience/giy084)
Supplement: Supplemental Figure [file giy084_supplemental_figure.pdf]

supplementary_figure_for_referees

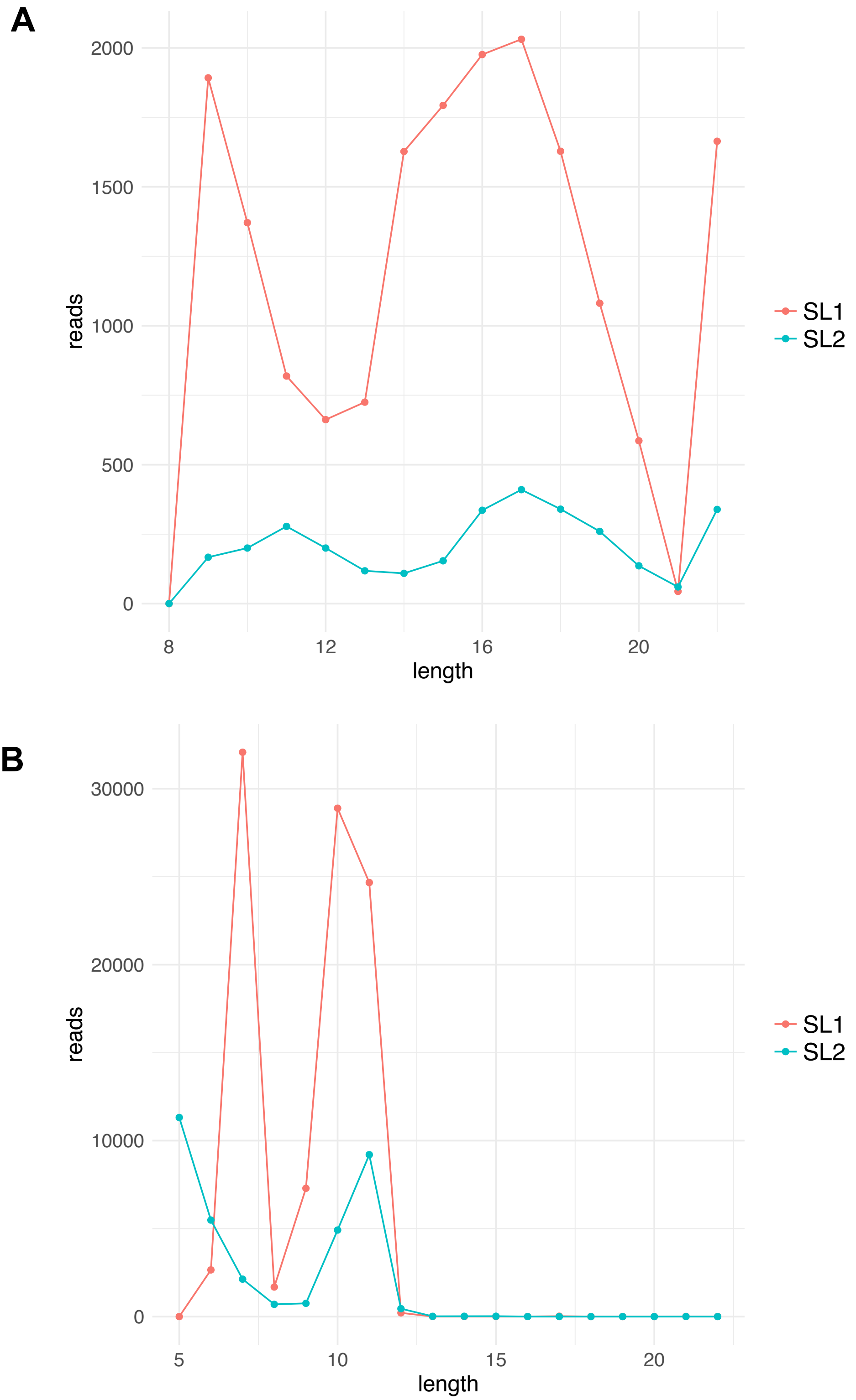

supplementary_figure_for_referees: A) Number of properly oriented significant alignments found by SL-quant on the *SRR2832497* dataset (single-end mode) by alignment length on the SL1 or SL2 sequences. **B)** Number of properly oriented significant alignments found by with the method used in *Tourasse et al, 2017* on the *SRR1585277* dataset by alignment length on the SL1 or SL2 sequences.
