# Supplementary material for: SL-quant: a fast and flexible pipeline to quantify spliced leader trans-splicing events from RNA-seq data
Source: Gigascience. 2018 Jul 11;7(7):giy084. doi: 10.1093/gigascience/giy084 (PMC6055573; doi:10.1093/gigascience/giy084)
Supplement: Response_to_Reviewer_Comments_Original_Submission.pdf [file giy084_response_to_reviewer_comments_original_submission.pdf]

Author's Response To Reviewer Comments

Close

GIGA-D-18-00139
SL-quant: A fast and flexible pipeline to quantify spliced leader trans-splicing events from RNA-seq data.
Carlo Yague-Sanz, M.D.; Damien Hermand, PhD
GigaScience

Dear Mr Yague-Sanz,

Your manuscript "SL-quant: A fast and flexible pipeline to quantify spliced leader trans-splicing events from RNA-seq data." (GIGA-D-18-00139) has been assessed by our reviewers. Based on these reports, and my own assessment as Editor, I am pleased to inform you that it is potentially acceptable for publication in GigaScience, once you have carried out some essential revisions suggested by our reviewers.

Their reports, together with any other comments, are below. Please also take a moment to check our website at https://giga.editorialmanager.com/ for any additional comments that were saved as attachments.

In addition, please register any new software application in the SciCrunch.org database to receive a RRID (Research Resource Identification Initiative ID) number, and include this in your manuscript. This will facilitate tracking, reproducibility and re-use of your tool.

Once you have made the necessary corrections, please submit a revised manuscript online at:

https://giga.editorialmanager.com/

If you have forgotten your username or password please use the "Send Login Details" link to get your login information. For security reasons, your password will be reset.

Please include a point-by-point within the 'Response to Reviewers' box in the submission system. Please ensure you describe additional experiments that were carried out and include a detailed rebuttal of any criticisms or requested revisions that you disagreed with. Please also ensure that your revised manuscript conforms to the journal style, which can be found in the Instructions for Authors on the journal homepage.

The due date for submitting the revised version of your article is 20 Aug 2018.

We look forward to receiving your revised manuscript soon.

Best wishes,

Nicole Nogoy, Ph.D
GigaScience
www.gigasciencejournal.com

>>> We thank the editor and reviewers for their insightful and constructive comments. We agree with reviewers 1 and 2 that the method should be validated on additional datasets. We therefore applied SL-quant on six unrelated RNA-seq libraries from a total of five different organisms. The results of this new analysis are shown in a new table 3. Beside this major change, we have responded to all comments raised by the referees as detailed in the point-by-point response below. The RRID SCR_016205 has been attributed to this work and is indicated in the 'Availability of supporting source code and requirements' section. The revised version of this manuscript is attached with the changes to the original submission marked in red for clarity.

Reviewer reports:
-Reviewer #1: In this study, Carlo and Damien developed a fast and flexible pipeline, SL-quant, to quantify spliced leader trans-splicing events from RNA-seq data. SL trans-splicing is prevalent in nematodes. Accurate and fast identification of SL trans-splicing is of importance to understand the molecular mechanism and biological significance of SL trans-splicing. The algorithm the authors proposed in this study can be applied to both paired-end and single-end RNA-seq data. And the pipeline accurately quantifies SL trans-splicing events.

It is overall a nice piece of technical note. The processing of the data seems really fast. My major concern is whether the authors could expand the species in which this pipeline can be applied to. SL trans-splicing is observed in many nematode species, and RNA-seq data are available in modENCODE. Comparative genomics analyses have been performed among these species (e.g. PMID: 22772596, 18218978). If this pipeline can be applied to other nematode species (especially C. briggsae, C. remanei, and C. brenneri, it would have a much greater impact.

>>> SL-quant can indeed be applied to species beyond C. elegans. It only requires (1) the genome sequence, (2) the SL sequences and (3) the gene annotation of the species. As a proof of concept, we have applied SL-quant to datasets from C. briggsae (the datasets from PMID 18218978), C. remanei (modENCODE_4206), C. brenneri (modENCODE_4705), and from the non-nematode T. brucei (SRR038724). As we could not find annotation for the SL sequences of C. briggsae and C. remanei, we used the set of consensus SL sequences for nematodes identified in (PMID 24130571) instead. The results of the SL trans-splicing quantification are summarized in a new table 3. Hundreds of thousands of trans-splicing events were identified and most of them originate from the expected consensus acceptor sites. This demonstrates that SL-quant can be applied to various species in the nematode phylum and beyond.

-A minor comment: The distribution of length in Figure 2C is a bit surprising. Can this be an artifact of the pipeline? I cannot think of any, but the authors may want to be more careful about it.

>>> The length of reads alignment to the SL-sequence depends on:
- Where the reverse transcription stops during the first strand synthesis of the library preparation. Does it reach the 5' end of the RNA fragment or is it blocked prematurely because of the cap or secondary structures?
- Where the second strand synthesis starts. In common RNA-seq library preparation, the

reaction is primed by RNA oligonucleotides generated by the digestion of the RNA-DNA duplex obtained after the first strand-synthesis. Therefore, the 5’ end of the final dsDNA is truncated.
- The minimal length for an alignment to be reported as significant by BLAST.
We assume that we are not finding many SL-sequences longer than 11 nt in the reads as a result of the first two points indicated above, while 9 nt appears to be the minimal length for an alignment to be significant in BLAST (with our settings, which are detailed in the method section). This issue is now more carefully explained in the manuscript. To further investigate this issue, we now applied SL-quant to an additional C. elegans dataset (SRR2832497) generated using an alternative library preparation protocol based on the direct ligation of adapters on the RNA, which ensures that the full RNA fragments are sequenced. Consistent with our interpretation, the distribution of read alignment length from this dataset ranges from 9 to 22 using the same BLAST settings (see the attached supplementary_figure_for_referees.pdf file, panel A).

-Do other methods have a similar results?

>>> Using the re-implementation of the method used in Tourasse et al., the minimal alignment length drops to 6 nt – as expected given the cutadapt parameters – but the distribution still peaks at 10-11 nt with almost no longer alignments (see the attached supplementary_figure_for_referees.pdf file, panel B). This is consistent with the idea that the length distribution is dependent on the data (presumably because of the library preparation), and not on the method.

Reviewer #2: Major comments:

-Paper derives the conclusion purely based on 2 samples. This is not enough to show the performance of the introduced method. Authors are encouraged to use simulated data and study the effect of read length, throughput and error rate on the accuracy of the proposed method.

>>> As stated in the response to the editor and reviewer 1, we agree that two samples is not enough. We have therefore analysed six additional samples from various organisms. Library preparation protocols, read length and throughput vary greatly in those datasets. This shows – perhaps more convincingly than it would with simulated reads – that SL-quant can be applied to a broad range of data. The results of the SL trans-splicing quantifications are summarized in the new table 3

-For the reader from the human genomics background it will be helpful if the authors provide the analogy with human trans splicing and fusion events, which are usually detected from RNA-Seq data. Explaining how the short spliced leader (SL) are related to human trans splicing and fusion events can be helpful. Authors can refer to this recent paper : Mangul, Serghei, et al. "ROP: Dumpster Diving in RNA-sequencing to find the source of 1 trillion reads across diverse adult human tissues." Genome biology 19.1 (2018): 36.

>>> We now mention in the manuscript that trans-splicing exists in human as well, although it happens at a low frequency.

-Proposed method can work on any BAM file with both mapped and unmapped reads. This is FANTASTIC, and I am so glad authors are aware of importance of re-suing previously

generated data. Is there a minimum read length, which method can handle? For example, will 50 bp be enough?

>>> We thank the referee for his/her enthusiasm. There is no real minimal read length for the method to work, but ideally, the read should be sufficiently long as to allow them to map uniquely after trimming of the SL-sequence. The number of nucleotides trimmed depends on the dataset (organism, library preparation, …) and can vary from read to read. Nevertheless, 50 nt reads are certainly enough in C. elegans. Indeed, we successfully applied SL-quant to the 41 nt (trimmed down to 19-32 nt) reads of the SRR2832497 dataset. The read length in the various datasets tested is now clearly stated in table 3.

-It is great to see that the proposed method can take the pairing information into account. Authors are recommended to comments how the proposed strategy compares to Nicolae, Marius, et al. "Estimation of alternative splicing isoform frequencies from RNA-Seq data." Algorithms for molecular biology 6.1 (2011): 9 in terms of modeling the fragment length distribution.

>>> The fragment length distribution is not directly modelled by SL-quant. In fact, pairing information is used at only two steps in paired-end mode: when the reads are being pre-filtered (fragment length is irrelevant here since only one mate is mapped) and during the remapping of the SL-containing reads by HISAT2. For this last step, we kept the default HISAT2 parameters regarding the maximum fragment length allowed for concordant mapping (ignoring introns), which is 500 nt.

-Section SL_containing reads identification. More details about the real data are needed. For example, how library were prepared? Number of mapped and unmapped reads? Read length?

>>> These are indeed important information that are now detailed in the methods section of the manuscript and table 3. The number of mapped reads is not explicitly indicated but can be inferred from the total number of reads and the number of input reads (= the number of unmapped reads in single-end mode).

-Effect of library preparation protocol needs to be discussed? For example polyA vs ribo depletion

>>> As stated above in the response to reviewer 1 (minor comment), the library preparation (random priming vs ligation method) is of great importance as it affects whether the 5' end of the SL-sequences can be included in the reads. This is now discussed more carefully in the "SL-containing reads identification" section. Concerning poly-A versus ribodepletion, we do not expect a dramatic impact because both protocols enrich for mRNA and SL-containing reads. To make sure of this, one should compare libraries made with either poly-A selection or ribodepletion from exactly the same RNA samples. To the best of our knowledge, such datasets are not available in C. elegans.

-Paper discuss the detection power of methods. It would be helpful if authors can define standard statistical measures. Such as PPV, Sensitivity and explain how they define TP, FP, FN

>>> Sensitivity, specificity, TP and FP are now clearly defined in the relevant sections of

the manuscript (see also the point below). We did not report PPV and therefore did not define it.

-It is not clear how Specificity is defined? To define Specificity one needs to define TN. It is not clear how TN are defined in this context

>>> In the context of the prediction of gene position in operon based on SL-quant results, the definition of TN is straightforward: it is a gene correctly called as not being in positions two and over within operons by our predictor. Specificity, TPR and FPR can therefore be calculated exactly as shown in the ROC curve (figure 4.B). However, when comparing the methods on their ability to call trans-splice sites, the concept of sensitivity becomes relative and cannot be precisely calculated as the truth is unknown. However, we used the following approximations: (1) Sensitivity (TN/N) can be expressed as [1-(FP/N)] because TN=(N-FP); (2) N (the number of negative calls) is approximated as constant because regardless of the method used and dataset studied, ~99.99% of the genome is called negative for trans-splice sites; (3) FP (the number of false positives) is approximated as genomic positions called trans-splice sites not bearing consensus sequences. According to those reasonable approximations, we consider a method “more specific” if it calls less non-consensus trans-splice sites than another method. The reviewer was right to point out this important issue, which is now more carefully explained in the manuscript.

-The authors have done great job documenting the method. However, it would be really helpful to provide full installation scripts. Which will provide one line solution to install all the dependencies. Right now the commands are provided for Mac only. The installation script can be based on conda install, pip or brew.

>>> Actually, the commands were provided for linux as well, based on linuxbrew, the linux equivalent of brew. While the installation is not a one-liner, we believe that it is sufficiently straightforward as to allow seamless installations of SL-quant for most users. Compiling the installation lines in one installation script can perhaps be dangerous if the user does not want to install some dependencies (for instance if he already has a brew-independent installation, this can lead to conflicts). We consider the current step-by-step approach of the installation safer for now. Nevertheless, we will keep the reviewer recommendation in mind for future development of the method and documentation.

Minor comments:
-Usually the blast is referred with all capitals i.e. BLAST. Also it needs to be clarified , if the original BLAST was used or megablast by BLAST+

>>> We used the BLAST+ suite that allows selection from a range of ‘tasks’ (megablast (default) ,blastn, blastn-short, …). Within SL-quant, the task ‘blastn’ is used. This has been clarified in the method section. BLAST is now written in capitals.

Reviewer #3:
The authors describe a method for analyzing unmapped RNA-seq reads to find signatures for trans-splicing. Reads are BLAST-aligned to the SL sequence and, where it is detected, it is trimmed off and the reads are realigned with HISAT2 and genes are quantified with featureCounts.

Results are validated by considering factors such as how specific the SL mappings are to the

3' end of the reads, whether expected motifs are present, and whether SL2 events seem specific to genes beyond the first in the operon.

The authors argue, and it is true as far as I could find, that this is the first software tool for automatically detecting trans-splicing from RNA-seq reads.

The software is well documented and is distributed under the MIT license.

Major comments:

-The authors argue that the tool is fast since it completes in 10 minutes. It would be helpful for the reader if this could instead be expressed as the number of reads (with unpaired and paired considered separately) processed per unit time. Otherwise it is hard to extrapolate from the 10-minute result. The number of reads in the two evaluated datasets should be reported.

>>> The number of input reads – the reads that are effectively blasted – is indicated in table 1 and the new table 3. About 106 reads are processed within five minutes. As this is an important point, we now indicate this information in the main text as well. Note that the speed per input read is not so great (the bottleneck being the BLAST step), but one of the major features of SL-quant that make it efficient is its pre-filtering step: only the reads likely to carry an SL sequence are taken as input by BLAST. The total number of reads in the evaluated datasets, which is much larger, is now also reported in the tables 1 and 3.

Minor comments:

-"Every run 5 completed within 10 min" -- the authors should clarify how many simultaneous threads were used. Based on the parameters listed in the Methods section, I think this result is using a single thread.

>>> We used the default options of SL-quant, which is 4 threads as detailed in the “advanced usage” section of our github page. This option can be modified according to the instruction on our github page (‘advanced usage’ section). We agree that is an important information and it has been clarified in the manuscript.

-"8 GO RAM" should presumably be "8 GB RAM"

>>>This was corrected accordingly.

Close
