# Supplementary material for: SL-quant: a fast and flexible pipeline to quantify spliced leader trans-splicing events from RNA-seq data
Source: Gigascience. 2018 Jul 11;7(7):giy084. doi: 10.1093/gigascience/giy084 (PMC6055573; doi:10.1093/gigascience/giy084)
Supplement: Reviewer_1_Report_(Original_Submission) -- Wenfeng Qian, PhD — 5/20/2018 Reviewed [file giy084_reviewer_1_report_(original_submission).pdf]

Reviewer Report

Title: **SL-quant: A fast and flexible pipeline to quantify spliced leader trans-splicing events from RNA-seq data.**

Version: **Original Submission** **Date:** 5/20/2018

Reviewer name: **Wenfeng Qian,**

Reviewer Comments to Author:

In this study, Carlo and Damien developed a fast and flexible pipeline, SL-quant, to quantify spliced leader trans-splicing events from RNA-seq data. SL trans-splicing is prevalent in nematodes. Accurate and fast identification of SL trans-splicing is of importance to understand the molecular mechanism and biological significance of SL trans-splicing. The algorithm the authors proposed in this study can be applied to both paired-end and single-end RNA-seq data. And the pipeline accurately quantifies SL trans-splicing events. It is overall a nice piece of technical note. The processing of the data seems really fast. My major concern is whether the authors could expand the species in which this pipeline can be applied to. SL trans-splicing is observed in many nematode species, and RNA-seq data are available in modENCODE. Comparative genomics analyses have been performed among these species (e.g. PMID: 22772596, 18218978). If this pipeline can be applied to other nematode species (especially C. briggsae, C. remanei, and C. brenneri, it would have a much greater impact. A minor comment:The distribution of length in Figure 2C is a bit surprising. Can this be an artifact of the pipeline? I cannot think of any, but the authors may want to be more careful about it. Do other methods have a similar results?

Level of Interest

Please indicate how interesting you found the manuscript: Choose an item.

Quality of Written English

Please indicate the quality of language in the manuscript: Choose an item.

Declaration of Competing Interests

Please complete a declaration of competing interests, considering the following questions:

- Have you in the past five years received reimbursements, fees, funding, or salary from an organisation that may in any way gain or lose financially from the publication of this manuscript, either now or in the future?
- Do you hold any stocks or shares in an organisation that may in any way gain or lose financially from the publication of this manuscript, either now or in the future?
- Do you hold or are you currently applying for any patents relating to the content of the manuscript?
- Have you received reimbursements, fees, funding, or salary from an organization that holds or has applied for patents relating to the content of the manuscript?
- Do you have any other financial competing interests?

- Do you have any non-financial competing interests in relation to this paper?

If you can answer no to all of the above, write 'I declare that I have no competing interests' below. If your reply is yes to any, please give details below.

I declare that I have no competing interests.

I agree to the open peer review policy of the journal. I understand that my name will be included on my report to the authors and, if the manuscript is accepted for publication, my named report including any attachments I upload will be posted on the website along with the authors' responses. I agree for my report to be made available under an Open Access Creative Commons CC-BY license (http://creativecommons.org/licenses/by/4.0/). I understand that any comments which I do not wish to be included in my named report can be included as confidential comments to the editors, which will not be published.

I agree to the open peer review policy of the journal

To further support our reviewers, we have joined with Publons, where you can gain additional credit to further highlight your hard work (see: https://publons.com/journal/530/gigascience). On publication of this paper, your review will be automatically added to Publons, you can then choose whether or not to claim your Publons credit. I understand this statement. Yes
