# Supplementary material for: SL-quant: a fast and flexible pipeline to quantify spliced leader trans-splicing events from RNA-seq data
Source: Gigascience. 2018 Jul 11;7(7):giy084. doi: 10.1093/gigascience/giy084 (PMC6055573; doi:10.1093/gigascience/giy084)
Supplement: Reviewer_2_Report_(Original_Submission) -- Serghei Mangul — 05/22/2018 Reviewed [file giy084_reviewer_2_report_(original_submission).pdf]

Reviewer Report

Title: SL-quant: A fast and flexible pipeline to quantify spliced leader trans-splicing events from RNA-seq data.

Version: Original Submission **Date:** 5/22/2018

Reviewer name: Serghei Mangul

Reviewer Comments to Author:

Major comments:-Paper derives the conclusion purely based on 2 samples. This is not enough to show the performance of the introduced method. Authors are encouraged to use simulated data and study the effect of read length, throughput and error rate on the accuracy of the proposed method. For the reader from the human genomics background it will be helpful if the authors provide the analogy with human trans splicing and fusion events, which are usually detected from RNA-Seq data. Explaining how the short spliced leader (SL) are related to human trans splicing and fusion events can be helpful. Authors can refer to this recent paper : Mangul, Serghei, et al. "ROP: Dumpster Diving in RNA-sequencing to find the source of 1 trillion reads across diverse adult human tissues." Genome biology 19.1 (2018): 36.-Proposed method can work on any BAM file with both mapped and unmapped reads. This is FANTASTIC, and I am so glad authors are aware of importance of re-suing previously generated data. Is there a minimum read length, which method can handle? For example, will 50 bp be enough?-It is great to see that the proposed method can take the pairing information into account. Authors are recommended to comments how the proposed strategy compares to Nicolae, Marius, et al. "Estimation of alternative splicing isoform frequencies from RNA-Seq data." Algorithms for molecular biology 6.1 (2011): 9 in terms of modeling the fragment length distribution. -Section SL_containing reads identification. More details about the real data are needed. For example, how library were prepared? Number of mapped and unmapped reads? Read length?-Effect of library preparation protocol needs to be discussed? For example polyA vs ribo depletion-Paper discuss the detection power of methods. It would be helpful if authors can define standard statistical measures. Such as PPV, Sensitivity and explain how they define TP, FP, FN-It is not clear how Specificity is defined? To define Specificity one needs to define TN. It is not clear how TN are defined in this context-The authors have done great job documenting the method. However, it would be really helpful to provide full installation scripts. Which will provide one line solution to install all the dependencies. Right now the commands are provided for Mac only. The installation script can be based on conda install, pip or brew. Minor comments:-Usually the blast is referred with all capitals i.e. BLAST. Also it needs to be clarified , if the original BLAST was used or megablast by BLAST+

Level of Interest

Please indicate how interesting you found the manuscript: Choose an item.

Quality of Written English

Please indicate the quality of language in the manuscript: Choose an item.

Declaration of Competing Interests

Please complete a declaration of competing interests, considering the following questions:

- Have you in the past five years received reimbursements, fees, funding, or salary from an organisation that may in any way gain or lose financially from the publication of this manuscript, either now or in the future?
- Do you hold any stocks or shares in an organisation that may in any way gain or lose financially from the publication of this manuscript, either now or in the future?
- Do you hold or are you currently applying for any patents relating to the content of the manuscript?
- Have you received reimbursements, fees, funding, or salary from an organization that holds or has applied for patents relating to the content of the manuscript?
- Do you have any other financial competing interests?
- Do you have any non-financial competing interests in relation to this paper?

If you can answer no to all of the above, write 'I declare that I have no competing interests' below. If your reply is yes to any, please give details below.

I declare that I have no competing interests

I agree to the open peer review policy of the journal. I understand that my name will be included on my report to the authors and, if the manuscript is accepted for publication, my named report including any attachments I upload will be posted on the website along with the authors' responses. I agree for my report to be made available under an Open Access Creative Commons CC-BY license (http://creativecommons.org/licenses/by/4.0/). I understand that any comments which I do not wish to be included in my named report can be included as confidential comments to the editors, which will not be published.

I agree to the open peer review policy of the journal

To further support our reviewers, we have joined with Publons, where you can gain additional credit to further highlight your hard work (see: https://publons.com/journal/530/gigascience). On publication of this paper, your review will be automatically added to Publons, you can then choose whether or not to claim your Publons credit. I understand this statement. Yes
