# Supplementary material for: SL-quant: a fast and flexible pipeline to quantify spliced leader trans-splicing events from RNA-seq data
Source: Gigascience. 2018 Jul 11;7(7):giy084. doi: 10.1093/gigascience/giy084 (PMC6055573; doi:10.1093/gigascience/giy084)
Supplement: Reviewer_3_Report_(Original_Submission) -- Ben Langmead — 22 May 2018 Reviewed [file giy084_reviewer_3_report_(original_submission).pdf]

Reviewer Report

Title: SL-quant: A fast and flexible pipeline to quantify spliced leader trans-splicing events from RNA-seq data.

Version: Original Submission **Date:** 22 May 2018

Reviewer name: Ben Langmead

Reviewer Comments to Author:

The authors describe a method for analyzing unmapped RNA-seq reads to find signatures for trans-splicing. Reads are BLAST-aligned to the SL sequence and, where it is detected, it is trimmed off and the reads are realigned with HISAT2 and genes are quantified with featureCounts.Results are validated by considering factors such as how specific the SL mappings are to the 3' end of the reads, whether expected motifs are present, and whether SL2 events seem specific to genes beyond the first in the operon.The authors argue, and it is true as far as I could find, that this is the first software tool for automatically detecting trans-splicing from RNA-seq reads. The software is well documented and is distributed under the MIT license.Major comments:The authors argue that the tool is fast since it completes in 10 minutes. It would be helpful for the reader if this could instead be expressed as the number of reads (with unpaired and paired considered separately) processed per unit time. Otherwise it is hard to extrapolate from the 10-minute result. The number of reads in the two evaluated datasets should be reported.Minor comments:"Every run 5 completed within 10 min" -- the authors should clarify how many simultaneous threads were used. Based on the parameters listed in the Methods section, I think this result is using a single thread."8 GO RAM" should presumably be "8 GB RAM"

Level of Interest

Please indicate how interesting you found the manuscript: Choose an item.

Quality of Written English

Please indicate the quality of language in the manuscript: Choose an item.

Declaration of Competing Interests

Please complete a declaration of competing interests, considering the following questions:

- Have you in the past five years received reimbursements, fees, funding, or salary from an organisation that may in any way gain or lose financially from the publication of this manuscript, either now or in the future?
- Do you hold any stocks or shares in an organisation that may in any way gain or lose financially from the publication of this manuscript, either now or in the future?
- Do you hold or are you currently applying for any patents relating to the content of the manuscript?
- Have you received reimbursements, fees, funding, or salary from an organization that holds or has applied for patents relating to the content of the manuscript?

- Do you have any other financial competing interests?
- Do you have any non-financial competing interests in relation to this paper?

If you can answer no to all of the above, write 'I declare that I have no competing interests' below. If your reply is yes to any, please give details below.

I declare that I have no competing interests

I agree to the open peer review policy of the journal. I understand that my name will be included on my report to the authors and, if the manuscript is accepted for publication, my named report including any attachments I upload will be posted on the website along with the authors' responses. I agree for my report to be made available under an Open Access Creative Commons CC-BY license (http://creativecommons.org/licenses/by/4.0/). I understand that any comments which I do not wish to be included in my named report can be included as confidential comments to the editors, which will not be published.

I agree to the open peer review policy of the journal

To further support our reviewers, we have joined with Publons, where you can gain additional credit to further highlight your hard work (see: https://publons.com/journal/530/gigascience). On publication of this paper, your review will be automatically added to Publons, you can then choose whether or not to claim your Publons credit. I understand this statement. Yes
